# How urban environments structure running behaviour in Beijing during winter, spring, and summer 2024: spatiotemporal patterns and configuration-specific interactions from running trajectory data

**DOI:** 10.1186/s13690-026-01919-x

**Published:** 2026-04-27

**Authors:** Cailin Qiu, Chendi Zhang, Ning Qiu, Xinyu Han, Tianjie Zhang

**Affiliations:** 1 School of Architecture and Urban Planning, Fuzhou University, Fuzhou, China; 2 Luskin School of Public Affairs, University of California, Los Angeles, USA; 3 School of Architecture and Urban Planning, Shandong Jianzhu University, Jinan, China; 4 Department of Urban and Rural Planning, School of Architecture, Tianjin University, Tianjin, China

**Keywords:** Urban running behaviour, Spatiotemporal patterns, Built environment, Interpretable machine learning, Health-supportive environments

## Abstract

**Background:**

Understanding how urban environments stimulate routine physical activity is a central issue in public health. Running, as a low-threshold and widely accessible exercise, serves as a sensitive indicator of environmental influence. Yet, few studies have used methods capable of capturing the non-linear and context-dependent interactions through which multiple built-environment factors jointly shape running. This study investigates the spatiotemporal patterns of running and identifies how combinations of environmental features across different urban scenarios affect behavioural activation.

**Methods:**

We analysed 83,302 GPS-tracked running trajectories from Beijing. Built-environment indicators were integrated across five dimensions and examined using Light Gradient Boosting Machine with SHapley Additive exPlanations (SHAP). To enhance behavioural interpretability, variables were grouped into scenario-informed contexts representing commuting, restorative, and training environments. Temporal and spatial analyses were also conducted to identify diurnal, weekly, and spatial clustering patterns of running activity.

**Results:**

Running exhibited clear morning-evening peaks, an inverted-U weekly rhythm, and a concentric spatial structure concentrated in central areas and along continuous spaces such as waterfronts and forest sports parks. SHAP interaction analysis further shows that non-linear built-environment effects are organised through configuration-specific interaction structures rather than marginal feature responses. Three dominant configurations are identified: (i) visual–landscape structures combining vegetation texture, sky openness, and water proximity; (ii) urban density configurations linking residential intensity, building form, accessibility, and nightlight intensity; and (iii) training configurations integrating facility density, route continuity, surface flatness, and shading.

**Conclusions:**

Running behaviour is jointly shaped by temporal rhythms, spatial clustering, and structured interactions among built-environment features. The findings demonstrate that physical activity patterns reflect the conditional contribution of co-occurring spatial attributes, providing evidence for designing more health-supportive and activity-friendly urban environments.

**Supplementary Information:**

The online version contains supplementary material available at 10.1186/s13690-026-01919-x.

**Table:** 

Text box 1. Contributions to the Literature
• This study shows that everyday running behaviour in cities is shaped by combinations of urban features rather than by single environmental factors acting alone.
• Using large-scale running trajectory data, the study demonstrates how urban environments jointly influence when and where people run in daily life.
• The findings indicate that supporting routine physical activity requires coordinated urban design rather than optimisation of individual environmental features.
• By linking urban form with population-level physical activity patterns, the study provides evidence to inform public health-oriented urban planning.

## Background

In recent years, global health challenges have intensified. According to the World Health Organization (WHO), physical inactivity has emerged as a leading risk factor contributing to the growing burden of non-communicable diseases, including cardiovascular conditions, diabetes, and depression [1]. As reported in 2020, more than a quarter of adults worldwide fail to meet recommended levels of physical activity [2]. In response, many countries have adopted the “Exercise is Medicine” initiative, promoting daily physical activity as an integral component of national health strategies [3]. Among various exercise forms, running stands out for its low entry barrier, widespread accessibility, and proven cardiovascular benefits, making it one of the most promising modes of everyday fitness [4]. In urban contexts, urban trails—characterised by green coverage, path continuity, and ease of access—have emerged as particularly conducive environments for running [5]. These trails have increasingly been recognised as vital components of urban health infrastructure [6].

A substantial body of research has confirmed that built environment characteristics significantly influence physical activity, particularly self-directed outdoor behaviours such as running and walking. Early studies primarily employed linear modelling approaches—such as OLS, logistic regression, structural equation modelling, and geographically weighted regression [5, 7]—to identify the effects of built environment features across dimensions including density (e.g. population, building) [8], accessibility (e.g. park proximity, metro distance) [9], and land-use mix (e.g. functional diversity) [10]. However, these studies often rely on small-scale ‘thick data’ derived from questionnaires and travel surveys, which introduces estimation bias and limits generalisability.

With the growing availability of running trajectory data and the application of machine learning techniques, an increasing number of studies have begun to explore the non-linear effects of the built environment on running behaviour. Recent systematic and integrative reviews have further consolidated this evidence, demonstrating that running behaviour exhibits distinct sensitivities to environmental exposures compared with general physical activity, particularly in relation to micro-scale street environments and route-level characteristics [11]. These approaches overcome the assumption of constant marginal effects inherent in traditional models, enabling the identification of threshold effects, U-shaped and inverse-U patterns, inflection points, and complex variable interactions [12, 13]. In particular, the integration of explainable algorithms such as SHAP and partial dependence plots (PDP) has significantly enhanced the transparency and interpretability of complex models [14]. Running-focused empirical studies have shown that environmental attributes such as enclosure, greenery, traffic exposure, and visual complexity shape not only running intensity but also perceived runnability and running pleasantness, with effects varying across urban contexts [15, 16]. These methodological advances have deepened our understanding of how built environment features influence running. For instance, population density may become a deterrent beyond a certain threshold due to perceived crowding and safety concerns, while the marginal benefits of green space tend to diminish, suggesting that moderate provision may better balance resource efficiency and behavioural incentives [17]. Nevertheless, most studies still rely on coarse spatial units such as TAZs, neighbourhoods, or buffer zones [18, 19], which fail to capture the micro-scale path spaces where running actually occurs. More critically, even when interaction terms are introduced [12], analyses often adhere to a unidirectional logic—examining how built environment features affect behaviour—without systematically considering the structural relationships among variables. Questions such as whether interaction structures are perceptible to users, whether they generate synergistic stimuli, and why they succeed in some contexts but not others remain largely unaddressed. While such research confirms the importance of environmental influences on running, it remains largely focused on identifying which variables matter [20], rather than how they operate in combination within specific spatial settings. This limited attention to the compositional logic and contextual adaptability of environmental factors constrains the theoretical development of spatial–behavioural relationships.

While explainable machine learning models have revealed complex non-linear relationships between variables and behaviour, most remain rooted in a variable–behaviour causality paradigm. They seldom provide a theoretical lens to interpret how environmental variables function collectively. In complex urban settings, behaviour is rarely a direct response to the presence or magnitude of a single factor; rather, it reflects an integrated reaction to structural configurations formed by multiple environmental elements within specific contexts [21]. Such structural synergies are neither incidental model outputs nor merely subjective perceptual responses—they may constitute fundamental mechanisms that trigger behavioural activation. Crucially, although affordance theory has evolved to address perceptual variation and contextual adaptability [22, 23], it continues to focus primarily on the fit between an individual and a single environmental feature. Much less attention has been paid to how multiple variables might interact to form structured configurations that are perceived, interpreted, and capable of eliciting behaviour. Therefore, a new theoretical framework is urgently needed—one that can explain which combinations of environmental variables, in which spatial contexts, elicit which types of behaviour.

To this end, this study uses large-scale running data to examine how multiple built-environment features jointly structure physical activity through configuration-specific interactions across different urban contexts. Drawing on 83,302 GPS-tracked trajectories from central Beijing, we integrated five dimensions of the built environment and applied interpretable machine learning to identify non-linear and configuration-dependent interaction structures. By modelling three typical running configurations—urban, restorative, and training—the analysis reveals distinct combinations of accessibility, landscape characteristics, and facility-related attributes that condition running intensit. These findings demonstrate how data from fitness applications can capture fine-grained behavioural dynamics and provide empirical evidence for understanding and designing health-supportive and activity-friendly urban environments.

## Methods

### Study area and units

**Fig. 1:**
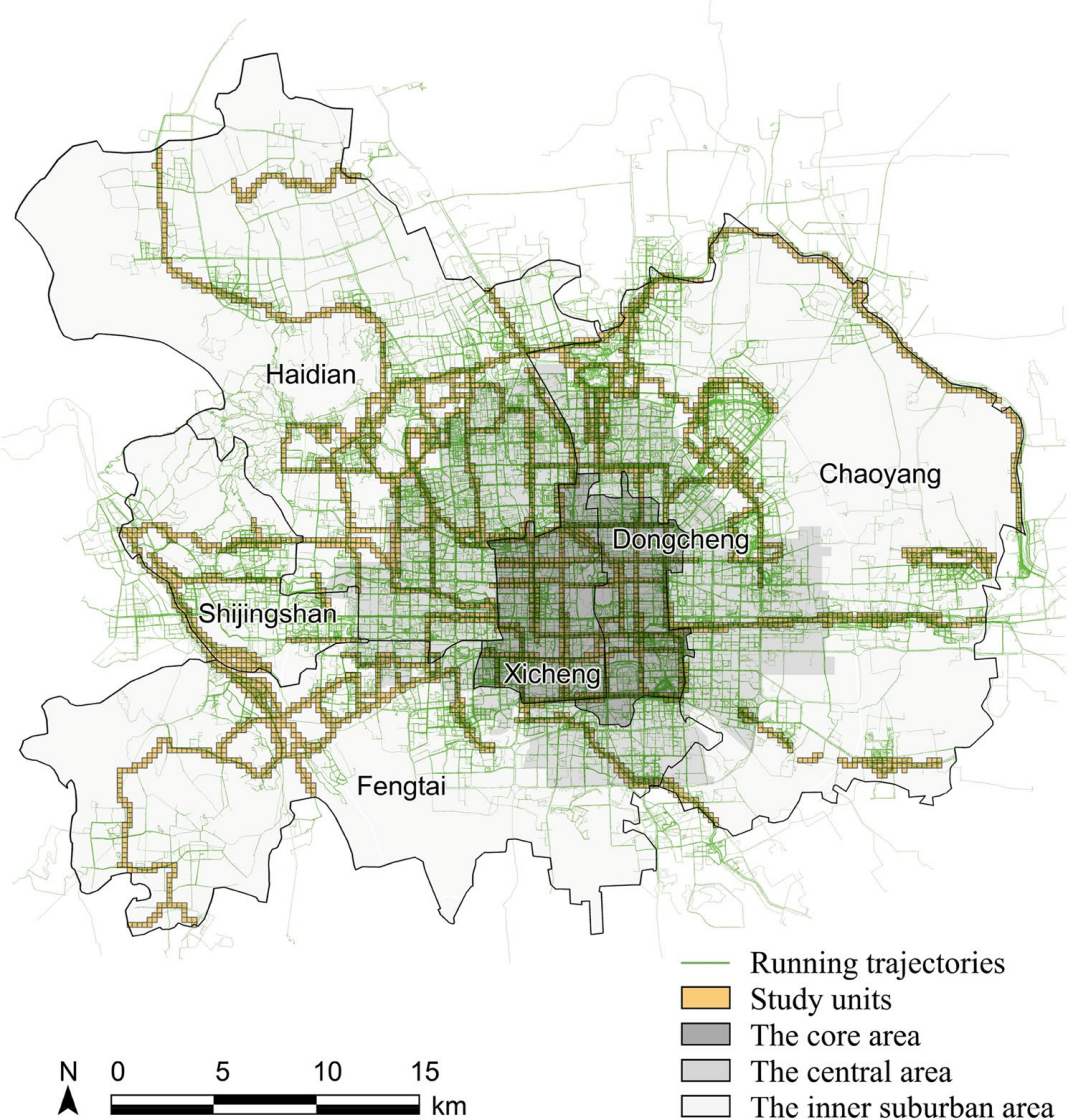
Study area and spatial distribution of running trajectories in the central urban area of Beijing during winter, spring, and summer 2024

This study is set in the central urban area of Beijing—an ideal context for examining running behaviour in dense metropolitan environments (Fig. 1). The area covers approximately 1,375.7 km² and houses over half of the city’s population. Characterised by high residential density, mature infrastructure, and functional diversity, it has seen sustained investment in urban trail development. Over 500 km of interconnected green corridors have already been established, with plans to expand the network to more than 750 km by 2035 [24]. The integration of green infrastructure and mobility systems renders this area exceptionally suited for continuous aerobic activity, making it a natural laboratory for studying recreational running in complex urban settings.

In this context, urban trails refer specifically to linear green spaces embedded within the built environment, designed to support walking, running, and cycling. While planning documents variously label them as “greenways”, “boulevard paths”, or “slow-traffic corridors”, these spaces share a multifunctional character that combines ecological value, active mobility, and recreational use [25]. Our definition of urban trails emphasises continuity, greenery, and accessibility—features that align closely with the everyday movement patterns of urban residents. As such, they offer a functionally and behaviourally meaningful spatial unit for modelling the interplay between environmental attributes and running activity.

To approximate environmental exposure during running as a continuous and mobile process, we adopted a trail-centred grid-based spatial unit, rather than conventional street-segment or residential buffer approaches. Urban trails function as linear movement corridors, along which runners experience sequential environmental cues during sustained motion. A 250 m × 250 m trail-centred grid was used as the primary analytical unit. This scale corresponds to approximately 1–1.5 min of continuous running, which aligns with the temporal window over which runners integrate environmental cues such as shading, enclosure, openness, and surface conditions [26]. Compared with finer spatial units, this scale better preserves exposure continuity along running routes and avoids excessive fragmentation of the movement context [27]. The 250 m grid is not treated as an optimal or exclusive scale. To assess scale sensitivity [28], we conducted supplementary analyses using a 500 m grid and compared key non-linear effects and interaction structures across scales (Supplementary Material S2). This analysis evaluates the robustness of identified structural patterns rather than selecting scales based on model fit [28]. Based on this definition, the study area was divided into 2,990 grid cells, each serving as the unit for environmental feature extraction and spatial modelling.

### Datasets

#### Running GPS trajectory data

This study draws on anonymised running trajectory data sourced from Keep, one of China’s most widely used fitness applications. The platform records its highest user engagement in Beijing, accounting for approximately 2.63% of the national user base [29], with most users under the age of 50 and a balanced gender distribution. Each entry contains key attributes including timestamp, GPS coordinates, running duration, average speed, and total distance. Although such datasets tend to over-represent younger, tech-savvy users, empirical validation demonstrates that crowdsourced platforms like Strava achieve high spatial precision (R²≈0.9 at monthly scale) in capturing actual recreational activity patterns, supporting their use for identifying environment and behaviour associations even if demographic biases exist [30].

To examine running behaviour on urban trails across different seasonal conditions [31], we compiled all running records in Beijing for February, May, and August 2024, covering winter, spring, and summer months. The initial dataset comprised 368,535 running trajectories. These records were then spatially filtered to retain only activities occurring within designated urban trails in the central urban area, resulting in 83,302 valid trajectories used for analysis. The spatial boundaries of urban trails were defined using official data from the Beijing Municipal Bureau of Landscape and Greening. Following established procedures [32], a 20-metre buffer was applied to the polyline network of urban trails to isolate trail-specific activity. Only trajectories entirely contained within this buffer zone were included. To ensure data integrity, all retained trajectories were manually reviewed to remove incomplete or misclassified entries.

#### Multi-source urban data

Built environment variables were derived from a range of high-resolution urban spatial datasets. Point-of-interest (POI) data for 2024 were accessed via the Amap API, covering 15 categories including public services, commercial and residential housing, retail, finance, education, healthcare, green spaces, government institutions, transport, and sports facilities. Road and river networks were extracted from OpenStreetMap (2024), while building footprints and storey information were drawn from the 2023 Amap AOI dataset for Beijing. Subdistrict-level population density data were sourced from Baidu’s population statistics platform. The shade index for walking and cycling routes was retrieved from Amap’s navigation service. In addition, Baidu Street View images were collected at 50-metre intervals across the central urban area on 20 July 2023, to support the extraction of visual and spatial attributes.

### Variables

#### Dependent variable

To quantify the spatial intensity of running behaviour within urban trails, the dependent variable was defined as the total trajectory length accumulated within each 250 m×250 m grid cell. For each GPS-tracked running trajectory, the segment length falling within each grid cell was calculated and aggregated across all trajectories to obtain cumulative running distance per cell. Compared with trajectory counts, cumulative trajectory length provides a more behaviourally meaningful measure of spatial activation, as it distinguishes locations dominated by frequent short loops from those embedded within longer running routes [15]. Given the pronounced right-skewed and long-tailed distribution of cumulative trajectory length, the dependent variable was transformed using log(1 + x) prior to modelling. This transformation preserves the relative ordering of spatial intensity while reducing the disproportionate influence of extreme values and improving robustness across grid cells.

#### Independent variables

To examine the spatial determinants of running flow—defined as the volume of running trajectories within each 250 m × 250 m grid—we adopted the widely used 5D framework. This framework classifies the built environment into five dimensions: Density, Diversity, Design, Destination accessibility, and Distance to transit [33]. It has been extensively applied in built environment and physical activity research, ensuring comparability with mainstream studies [34].

We constructed 18 indicators tailored to urban running. For Density, variables included Population Density (PopDen), Residential Density (ResDen), Building Density (BldDen), and Indoor Sports Facility Density (SportDen), reflecting user concentration and built intensity [8, 10, 33, 35]. Diversity was measured via Land Use Mix (LUM) and Vegetation Type Diversity (VTD), which represent spatial heterogeneity and ecological richness affecting sensory quality [10]. The Design dimension comprised six indicators of physical comfort and visual openness: Green Coverage Ratio (GreenRate), Green View Index (GVI), Shading Ratio (Shade), Sky View Factor (SVF), Surface Flatness (SurfFlat), Urban Trail Accessibility (UTAcc), Night-time Light Intensity (NTL) [36], and Urban Trail Continuity (Continuity) [7, 15, 37]. In particular, Surface Flatness is used to capture the evenness of pavement as a proxy for both comfort and safety, as uneven surfaces increase tripping and injury risk. NTL reflects nighttime illumination conditions, while Continuity measures network-based trail connectedness enabling continuous running. For Destination accessibility, we included Park Accessibility (ParkAcc) and Distance to Waterway (DistWater), capturing the appeal of naturalistic features [12, 17]. Finally, Distance to transit was represented by Intersection Density (IntNum) and Distance to Metro/Bus/Parking Entrance (DistTransit), reflecting network connectivity and route complexity [12, 38]. All variables were calculated using GIS techniques within the 250 m trail-centred grid cells. To ensure comparability, all indicators were standardised using Z-score transformation prior to modelling. Full definitions, computational formulas, and measurement units are presented in Table 1.

**Table 1:** Definitions and summary statistics of built-environment indicators used to analyse running behaviour in Beijing during winter, spring, and summer 2024

Variables (Abbr., Unit)	Description	Mean	Standard deviation
Density			
Population density (PopDen, people/km2)	Mean population within a 15-min walk buffer (based on gridded population data).	16113.946	9130.480
Residential Density (ResDen, m2/grid)	Residential land area within 15-min walk buffer.	54763.567	55554.481
Building Density (BldDen)	Total building footprint area per 250 m grid.	0.120	0.113
Indoor Sports Facility Density (SportDen)	Kernel density of indoor sports POIs (e.g. gyms, arenas).	17719.172	16077.076
Diversity			
Land Use Mix (LUM)	Entropy-based land-use diversity across POI categories with per 250 m grid.$\:\mathrm{L}\mathrm{U}\mathrm{M}=-\sum\:_{\mathrm{i}=1}^{\mathrm{n}}{\mathrm{p}}_{\mathrm{i}}\mathrm{*}\mathrm{l}\mathrm{n}\left({\mathrm{p}}_{\mathrm{i}}\right)/\mathrm{l}\mathrm{n}\left(\mathrm{n}\right)$ where$\:{\mathrm{p}}_{\mathrm{i}}$is the proportion of POI type$\:\mathrm{i}$, and$\:\mathrm{n}$is the number of POI categories.	0.454	0.570
Vegetation Type Diversity (VTD)	Shannon index of NDVI-based vegetation types. NDVI values were reclassified into five types: (1) Non-vegetated (excluded from analysis); (2) Sparse vegetation (NDVI 0.1–0.3), (3) Moderate vegetation (0.3–0.6), (4) Dense vegetation (0.6–0.8), and (5) Very dense vegetation (> 0.8).$\:\mathrm{V}\mathrm{T}\mathrm{D}=-\sum\:_{\mathrm{i}=1}^{\mathrm{k}}{\mathrm{p}}_{\mathrm{i}}\mathrm{*}\mathrm{l}\mathrm{n}\left({\mathrm{p}}_{\mathrm{i}}\right)/\mathrm{l}\mathrm{n}\left(\mathrm{n}\right)$ where$\:{\mathrm{p}}_{\mathrm{i}}$is the proportion of vegetation type$\:\mathrm{i}$.	0.370	0.254
Design			
Green Coverage Ratio (GreenRate, %)	Proportion of green-covered surfaces per grid.	0.385	0.252
Green View Index (GVI, %)	Average green pixel ratio in street view imagery, obtained via image semantic segmentation [39].	0.133	0.090
Shading Ratio (Shade, %)	Proportion of shaded segments based on Amap summer sun simulation.	0.439	0.183
Sky View Factor (SVF)	Openness of the sky calculated using a raster-based sky obstruction model with building height information and hemispherical sampling.	0.847	0.122
Surface Flatness (SurfFlat)	Manual pavement quality score (0–3) from imagery. 0: Severely uneven or damaged pavement. 1: Mostly flat with minor unevenness in parts. 2: Flat surface without dedicated jogging lanes or markings. 3: High-quality, smooth surface with professionally built running tracks.	2.355	0.715
Urabn Trails Accessibility (UTAcc)	Binary indicator of 15-min network walk access to urban trails using ArcGIS 10.8.	0.682	0.320
Night-time light intensity (NTL)	Represents ambient artificial illumination within each spatial unit, serving as a proxy for nighttime visibility and perceived safety conditions.	5499.375	197.698
Urban Trail Continuity (Continuity)	Quantifies the network-based connectedness of urban trail segments, capturing the degree to which the trail system supports uninterrupted running movement.	0.687	0.274
Destination accessibility			
Park Accessibility (ParkAcc, km2)	Gravity-based score considering park area and network distance.$$\:\begin{array}{c}{\mathrm{A}}_{\mathrm{i}}={\mathrm{E}}_{\mathrm{j}}{\mathrm{e}}^{-{\upbeta\:}{\mathrm{x}}_{\mathrm{i}\mathrm{j}}}\#\end{array}$$ where$\:{\mathrm{A}}_{\mathrm{i}}$denotes the accessibility of grid$\:\mathrm{i}$;$\:{\mathrm{x}}_{\mathrm{i}\mathrm{j}}$is the shortest network distance between grid$\:\mathrm{i}$and park$\:\mathrm{j}$;$\:{\mathrm{E}}_{\mathrm{j}}$is the attractiveness of park$\:\mathrm{j}$, measured by its area; and$\:{\upbeta\:}$is the impedance parameter empirically calibrated.	0.987	1.396
Distance to Waterway (DistWater, m)	Euclidean distance to nearest river/canal from each grid.	397.690	497.824
Distance to transit			
Number of Intersections (IntNum)	Total number of road intersections per grid.	0.361	0.563
Distance to Metro/Bus/Parking Entrance (DistTransit, m)	Euclidean distance to nearest metro/bus/parking entrance from grid centroid.	190.494	199.111

Note: All abbreviations used for built-environment indicators are explained in the table notes

### Study design

In urban built environments, running behaviour rarely arises from any single environmental attribute [23]; rather, it emerges from the runner’s integrated appraisal of multiple, concurrent environmental cues through perception–action coupling and multisensory experience [40]. In dynamic and mobile settings, the co-occurrence of visual, auditory, and tactile stimuli forms a multimodal sensory structure, within which behavioural responses become increasingly context-sensitive [41]. Capturing such perception–action pathways therefore requires analytical approaches capable of representing non-linear relationships and interaction structures while retaining interpretability.

To address this requirement, we deliberately avoid estimating a single comprehensive “kitchen-sink” model that aggregates all environmental variables. Instead, we adopt an interpretability-oriented configurational modelling strategy based on feature configuration analysis, in which parallel models are estimated on the same dataset using different configurations of environmental variables. By structuring variables into coherent feature spaces, this approach reduces the masking of non-linear effects, facilitates the identification of higher-order interaction structures, and aligns with established practices in interpretable machine learning that emphasise interaction-aware and configuration-based analysis [42, 43]. Importantly, these feature configurations do not classify individuals, label trajectories, or partition the sample; rather, they serve as analytical devices for organising environmental variables around distinct perceptual and functional emphases documented in environmental psychology and spatial behaviour research [44, 45]. From a methodological perspective, comparing multiple theoretically plausible feature configurations estimated on the same outcome is a well-established strategy for addressing specification uncertainty and enhancing interpretability, rather than assuming a single “correct” model specification [46, 47].

Within this framework, the analysis focuses on identifying structural affordance patterns, defined as recurrent and interpretable interaction structures among environmental variables that emerge consistently under a given feature configuration. This conception is grounded in ecological psychology, where affordances are understood not as isolated environmental attributes but as relational action possibilities arising from structured ensembles of environmental features relative to an actor’s capabilities [23]. Structural affordance is therefore not introduced as a new affordance theory; instead, it functions as an empirical descriptor of configuration-level affordance manifestations, capturing how multiple environmental cues are co-activated and jointly shape behavioural potential within specific spatial contexts. This perspective is consistent with prior work emphasising that affordances are context-dependent and realised through the co-presence of multiple environmental conditions rather than through single features in isolation [48, 49]. Accordingly, the analytical emphasis is placed on variable co-activation and interaction structures, rather than on categorical classification or the validation of pre-defined behavioural contexts.

The analysis is conducted using three alternative feature configurations, hereafter denoted by the shorthand labels Restorative, Urban, and Training. These labels are used solely to distinguish different configurations of environmental variables and do not represent behavioural categories, running types, or empirically observed contexts. The Restorative configuration, emphasising nature exposure and spatial openness, includes vegetation diversity (VTD), green coverage (GreenRate), green view index (GVI), sky view factor (SVF), park accessibility (ParkAcc), and distance to water (DistWater). The Urban configuration, emphasising accessibility, connectivity, and ambient urban intensity, includes population density (PopDen), residential density (ResDen), building density (BldDen), land-use mix (LUM), urban trail accessibility (UTAcc), distance to public transit (DistTransit), and night-time light intensity (NTL), used as a proxy for ambient artificial illumination and nighttime visual conditions at the spatial-unit level [36]. The Training configuration, emphasising functional and performance-related conditions, includes indoor sports facility density (SportDen), shading ratio (Shade), surface flatness (SurfFlat), intersection density (IntNum), and urban trail continuity (Continuity), a computed indicator capturing the structural continuity and network integration of running routes [15].

All models are estimated using the same dataset and the same outcome variable. SHAP values and SHAP interaction values are employed to quantify both local and global non-linear contributions, as well as pairwise interaction effects, within each feature configuration [42, 43]. Notably, structural affordance patterns may recur across different feature configurations, indicating that the proposed configurational modelling framework is capable of revealing emergent and reproducible interaction structures rather than merely reflecting the initial grouping of variables.

### Spatiotemporal patterns analysis

#### Temporal pattern analysis

To characterise the temporal dynamics of running behaviour, this study employed a multi-period harmonic regression model based on Fourier expansion. This approach identifies latent intra-day and intra-week periodic components from continuous time series data. Running is a typically cyclical activity influenced by both individual routines and social time schedules, thus exhibiting inherent daily and weekly recurrence patterns. The harmonic model allows these periodic components to be automatically detected from the time series without setting arbitrary thresholds. Moreover, it enables the superposition and interaction of periodic signals at different temporal scales (e.g., 24-hour and 7-day cycles), thereby capturing the coupling relationships between multiple behavioural rhythms in a systematic manner. Let $\:{y}_{t}$denote the running volume at time $\:t$; the model is specified as follows:

$${y}_{t}=\alpha\:+\sum\:_{k=1}^{{K}_{1}}\left[{\alpha\:}_{k}\mathrm{sin}\left(\frac{2\pi\:kt}{24}\right)+{b}_{k}\mathrm{cos}\left(\frac{2\pi\:kt}{24}\right)\right]+\sum\:_{m=1}^{{K}_{2}}\left[{c}_{m}\mathrm{sin}\left(\frac{2\pi\:mt}{168}\right)+{d}_{m}\mathrm{cos}\left(\frac{2\pi\:mt}{168}\right)\right]+\sum\:_{k,m}{\gamma\:}_{km}sin\left(\frac{2\pi\:kt}{24}\right)\mathrm{sin}\left(\frac{2\pi\:mt}{168}\right)+{\epsilon\:}_{t}$$

In this formulation, the first component represents the intra-day rhythm (24-hour cycle), capturing the morning–evening training structure; the second component describes the intra-week rhythm (168-hour, or 7-day cycle), reflecting differences between weekdays and weekends; and the third component, an interaction term, captures the modulation of the daily rhythm amplitude by the weekly rhythm. The harmonic orders are set to $\:{K}_{1}=12$and $\:{K}_{2}=4$, which are sufficient to capture the main waveform variations. $\:\alpha\:$denotes the intercept term, and $\:{\epsilon\:}_{t}$represents the random disturbance term.

To correct the skewness of the data and stabilise variance, the dependent variable $\:{y}_{t}$was transformed using $\:\mathrm{l}\mathrm{o}\mathrm{g}(1+{y}_{t})$. To mitigate multicollinearity among polynomial terms and ensure model smoothness, ridge regression (Ridge regularisation) was applied for parameter estimation. The optimisation function is defined as: $\:\genfrac{}{}{0pt}{}{min}{\beta\:}\lVert{y-X\beta\:\lVert}^{2}+\lambda\:\lVert{\beta\:\lVert}^{2}$, where $\:\lambda\:=1.0$is the regularisation coefficient that constrains the magnitude of the coefficients and suppresses high-frequency oscillations.

After model training, predicted values were generated over a high-density temporal grid and smoothed using a moving-average filter to obtain continuous temporal trends. The daily-aggregated data were subsequently fitted with a 7-day harmonic model ($\:{K}_{7}=4$) to extract the smooth structure of intra-weekly variation:

$${y}_{d}=\alpha\:+\sum\:_{k=1}^{{K}_{7}}\left[{\alpha\:}_{k}sin\left(\frac{2\pi\:kt}{7}\right)+{b}_{k}cos\left(\frac{2\pi\:kt}{7}\right)\right]+{\epsilon\:}_{d}$$

#### Spatial hot spot analysis

To identify the spatial clustering characteristics of running behaviour, this study conducted a hotspot–coldspot analysis based on the total length of running trajectories, defined as the total distance of each individual running session. The analysis was performed in ArcGIS 10.8, using the Getis–Ord Gi* statistic to detect statistically significant high-value (hotspot) and low-value (coldspot) areas. This approach was applied in a recent study on running and the built environment to reveal statistically significant spatial concentrations of running-related activity and environmental suitability [16]. Trajectory length was regarded as a composite indicator reflecting both exercise intensity and route selection preference, providing a more accurate representation of each area’s attractiveness to running activities than trajectory counts alone. Significant clusters were identified as high-value clusters (Z > 1.65, *p* < 0.1) and low-value clusters (Z<–1.65, *p* < 0.1).

### Non-linear analysis

#### LightGBM for interaction detection

To capture non-linear interaction mechanisms among multiple environmental variables, this study employs the Light Gradient Boosting Machine (LightGBM) to estimate a global response model, combined with SHAP (Shapley Additive Explanations) for interaction analysis [50]. This modelling framework focuses on how running behaviour responds to configuration-specific combinations of built environment features, with particular emphasis on marginal contributions and interaction structures across alternative feature configurations. LightGBM, a decision-tree-based ensemble learning algorithm, models complex non-linear relationships by iteratively fitting residuals in an additive framework. The objective is to minimise the following regularised loss function:

3 $$\mathrm{L}=\sum_{\mathrm{i}=1}^{\mathrm{n}}\ell\left({\mathrm{y}}_{\mathrm{i}},\mathrm{f}\left({\mathrm{x}}_{\mathrm{i}}\right)\right)+{\Omega\:}\left(\mathrm{f}\right)$$

where $\:{\mathrm{y}}_{\mathrm{i}}$ denotes the actual running flow in grid $\:\mathrm{i}$, $\:{\mathrm{x}}_{\mathrm{i}}$ is the corresponding vector of built environment variables (e.g. PopDen, SVF, GVI), $\:\mathrm{f}\left({\mathrm{x}}_{\mathrm{i}}\right)$ is the model prediction, $\:\mathcal{l}\left({\mathrm{y}}_{\mathrm{i}},\mathrm{f}\left({\mathrm{x}}_{\mathrm{i}}\right)\right)$ represents the squared loss, and $\:{\Omega\:}\left(\mathrm{f}\right)$ is a regularisation term controlling model complexity to avoid overfitting. Modelling was implemented using the LightGBM package in Python, with key parameters set as: n_estimators = 300, max_depth = 6, and learning_rate = 0.05. Five-fold cross-validation was applied to evaluate model performance.

#### SHAP-based interpretation of spatial and interaction effects

To improve the interpretability of model outputs, this study applies SHAP to both the LightGBM models. SHAP provides a consistent framework for quantifying the contribution of each built environment variable to the predicted running flow and for identifying interaction effects between variables. As a model-agnostic approach, SHAP supports both global importance ranking and local instance-level explanation, making it one of the most widely adopted interpretability tools in machine learning.

SHAP decomposes model predictions into additive marginal contributions of each input variable. For a given feature $\:\mathrm{i}$, its Shapley value $\:{{\varnothing}}_{\mathrm{i}}$ is defined as:

4 $${{\varnothing}}_{\mathrm{i}}=\sum_{\mathrm{S}\subseteq\:\mathrm{N}\left\{\mathrm{i}\right\}}\frac{\left|\mathrm{S}\right|!\left(\left|\mathrm{N}\right|-\left|\mathrm{S}\right|-1\right)!}{\left|\mathrm{N}\right|!}\left[\mathrm{f}\left(\mathrm{S}\cup\:\left\{\mathrm{i}\right\}\right)-\mathrm{f}\left(\mathrm{S}\right)\right]$$

where $\:\mathrm{N}$ denotes the full set of input features, $\:\mathrm{S}$ is a subset excluding $\:\mathrm{i}$, and $\:\mathrm{f}\left(\mathrm{S}\right)$ is the model prediction using features in $\:\mathrm{S}$. This enables the expression of the model output $\:\mathrm{g}\left({\mathrm{z}}^{{\prime\:}}\right)$ as:

5 $$\mathrm{g}\left({\mathrm{z}}^{{\prime\:}}\right)={{\varnothing}}_{0}+\sum_{\mathrm{i}=1}^{\mathrm{M}}{{\varnothing}}_{\mathrm{i}}{\mathrm{z}}_{\mathrm{i}}^{{\prime\:}}$$

where $\:{\mathrm{z}}_{\mathrm{i}}^{{\prime\:}}\in\:\left\{\mathrm{0,1}\right\}$ indicates whether feature $\:\mathrm{i}$ is present, $\:{{\varnothing}}_{0}$ is the baseline output, and $\:\mathrm{M}$ is the total number of features.

## Results

### Spatiotemporal patterns of running activity

The analysis is based on running trajectory data collected in February, May, and August 2024, covering both thermally adverse periods (winter cold and summer heat) and a climatically favourable period for outdoor exercise. To characterise fine-grained temporal rhythms under relatively unconstrained conditions, the detailed intra-month and intra-day analyses presented here focus on May, which represents the most suitable season for running in Beijing.

**Fig. 2:**
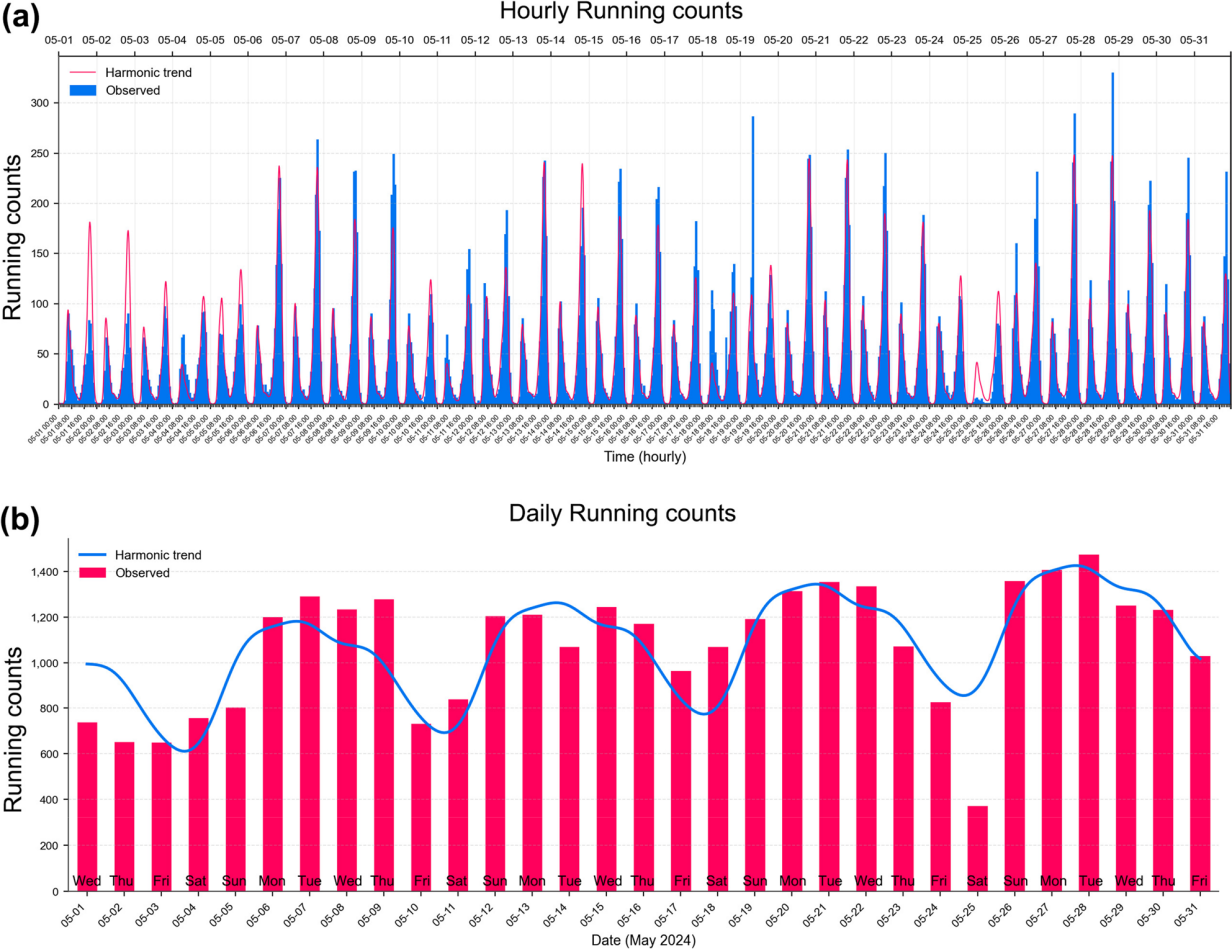
Temporal patterns of running behaviour in Beijing in May 2024. **a** Hourly running flow with harmonic fitting, showing two stable peaks in the morning and evening. **b** Daily running flow exhibiting an inverted-U pattern, increasing from Sunday to mid-week and declining towards Saturday

Figure 2a illustrates the hourly distribution and fitted trend of running behaviour from 1 to 31 May 2024. Overall, running activity exhibits a pronounced daily periodic pattern, characterised by two distinct peaks each day. The morning peak typically occurs between 07:00 and 09:00, while the evening peak is concentrated between 18:00 and 21:00, corresponding to the typical morning and evening exercise periods. This pattern is observed during the climatically most favourable month, allowing temporal regularities to be interpreted with minimal interference from extreme temperature constraints. The bimodal structure remains highly consistent throughout the month, suggesting that running behaviour is jointly constrained by daily routines and climatic conditions, thereby showing a strong rhythmic feature.

At the daily scale (Fig. 2b), running volume displays a clear intra-week fluctuation, following an approximate inverted U-shaped trend. Specifically, the volume gradually increases from Sunday to midweek (Tuesday and Wednesday), reaching its peak in the middle of the week, and then declines towards Saturday, when the lowest level is observed. The marked reduction during weekends—particularly on Saturdays—indicates that running activity is partially substituted by travel or other leisure activities during rest days, whereas weekdays maintain higher levels of exercise participation. By focusing on May, these weekly dynamics are less likely to be confounded by seasonal avoidance behaviour observed during periods of thermal stress, thereby providing a clearer representation of social-time modulation effects. In sum, running behaviour exhibits both intra-day rhythmicity and intra-week periodicity. The former reflects physiological routines, while the latter highlights the modulating influence of social time structures on exercise behaviour.

**Fig. 3:**
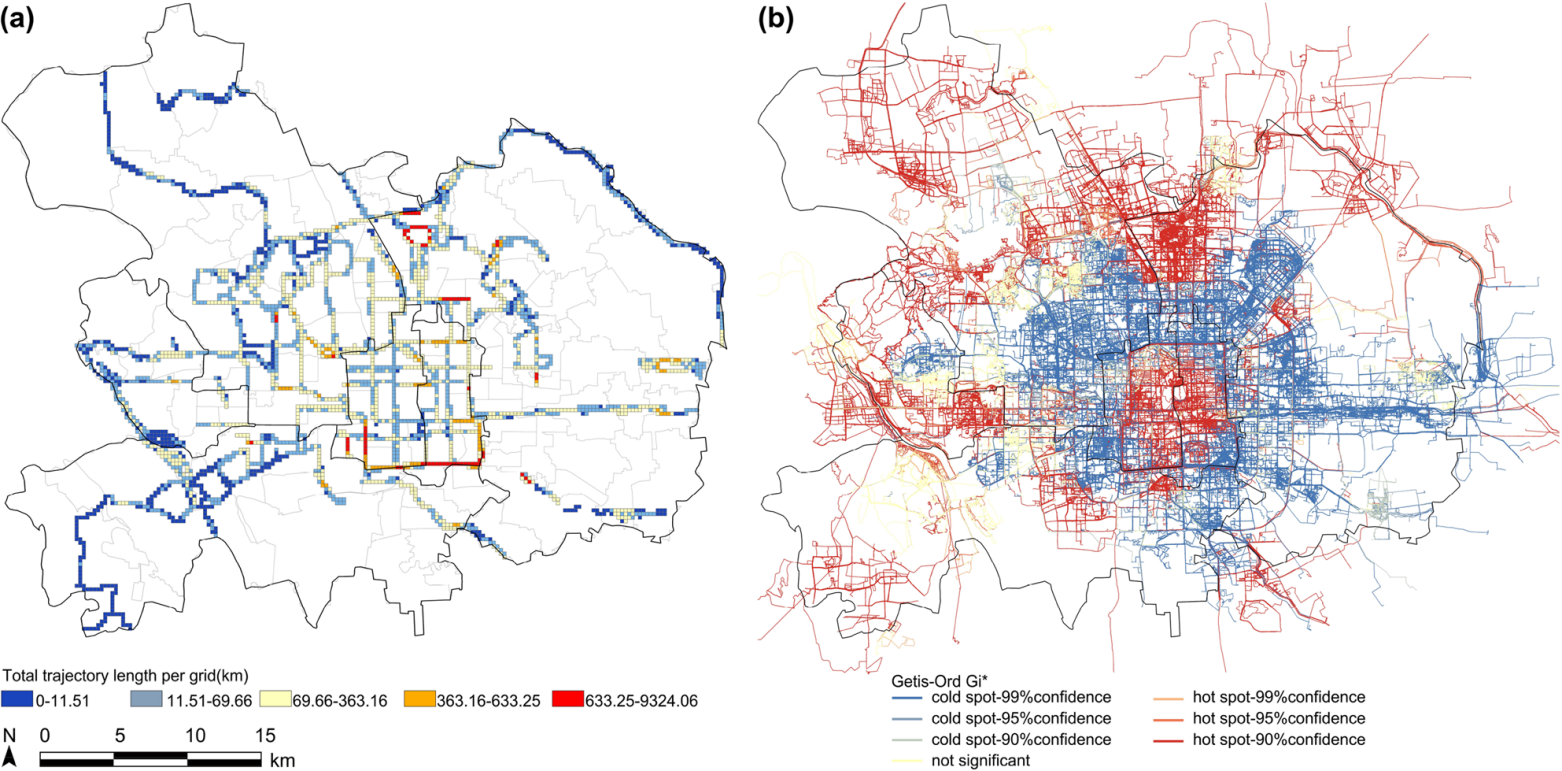
Spatial distribution of running activity in Beijing during winter, spring, and summer 2024. **a** Log-transformed total trajectory length, showing a concentric pattern with high values in the urban core. **b** Hot-spot analysis of trajectory length, identifying clusters along urban and suburban waterfront trails

Figure 3 illustrates the spatial distribution of running activity across Beijing. Figure 3a shows the distribution of total trajectory length per grid cell using the natural breaks classification, revealing a clear centre–periphery gradient, with higher running intensity concentrated in the central urban area and lower values in peripheral zones. High-intensity areas are primarily associated with waterfront fitness trails in the central city and circular running tracks within large forest sports parks, both of which support continuous and sustained running. In contrast, low-intensity areas are mainly located along ecological waterfront and forest-based urban trails in the urban periphery, where cumulative running distance remains limited.

Figure 3b presents the hotspot–coldspot analysis based on trajectory length. While the overall hotspot pattern largely mirrors the centre–periphery structure, additional hotspots emerge along suburban waterfront fitness trails, indicating their suitability for long-distance running. Cold spots are mainly observed in transitional zones between the urban core and the periphery, where fragmented road networks and mixed urban functions constrain route continuity. Overall, running activity exhibits a structured spatial pattern characterised by central concentration and secondary aggregation along ecological corridors and park systems.

### Configuration-specific feature importance and nonlinear association interpretation

Model performance was comparable across configurations (Restorative: R²=0.438, RMSE = 0.838, MAE = 1.108; Training: R²=0.430, RMSE = 1.683, MAE = 1.109; Urban: R²=0.437, RMSE = 1.540, MAE = 1.101). From a behavioural perspective, Restorative running is expected to be more environmentally sensitive (e.g., to perceived comfort and spatial quality), whereas Training running is more strongly constrained by individual goals and planning, making it less fully captured by built-environment features alone. Urban running combines routine leisure and everyday activity motives and therefore exhibits explanatory power closer to Restorative, and slightly higher than Training, under the same conservative modelling setup. Despite similar overall fit, SHAP analyses revealed distinct configuration-specific interaction structures, indicating that the configurational modelling framework captures structural interaction patterns beyond overall variable importance.

#### Feature importance across running configurations

**Fig. 4:**
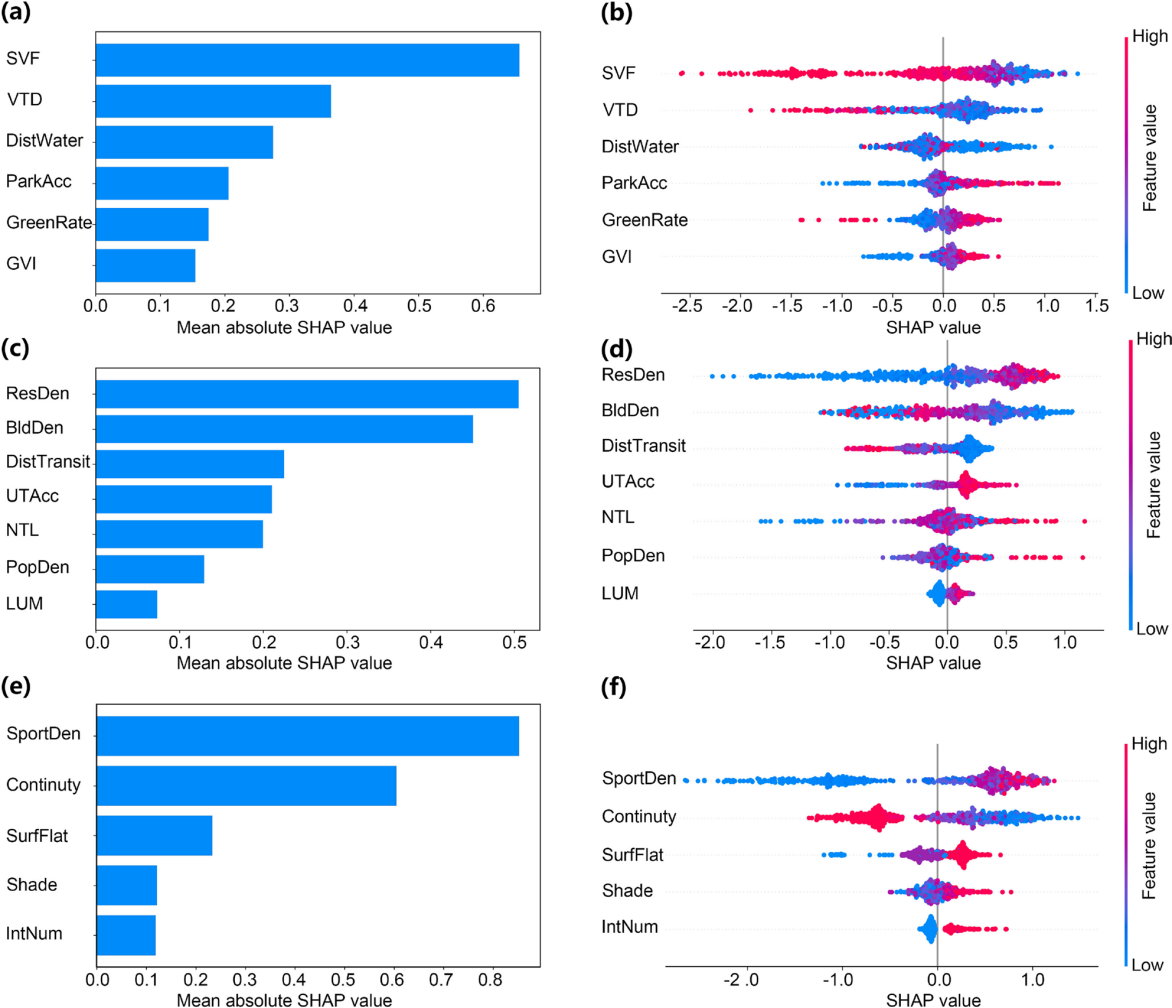
Relative importance of built-environment variables and distribution of Shapley additive explanations values for predicting running flow in Beijing during winter, spring, and summer 2024. (**a**/**c**/**e**) Mean absolute Shapley additive explanations (SHAP) values reflecting the overall contribution of each variable to model prediction. (**b**/**d**/**f**) SHAP summary plot illustrating the direction and magnitude of each variable’s effect. Warmer colours indicate higher feature values; cooler colours indicate lower values

Figure 4 summarises the relative importance and SHAP effect patterns of built environment variables across the restorative, urban, and training running configurations.

Under the restorative configuration (Fig. 4a–b), SVF is the most influential variable; however, its SHAP distribution reveals a clear inverse pattern, with higher values predominantly associated with negative SHAP values and lower values contributing positively to running flow. VTD shows a similar inverse tendency, where lower values are more frequently linked to positive SHAP contributions, while higher values cluster near zero or negative effects. DistWater exhibits a clear positive association at lower values, with blue points concentrated in the positive SHAP range, indicating that proximity to water bodies enhances running flow. ParkAcc, GreenRate, and GVI display weaker but predominantly positive contributions at higher values.

In the urban configuration (Fig. 4c–d), density-related variables dominate. Higher ResDen is strongly associated with positive SHAP values, whereas higher BldDen corresponds to negative contributions. Greater DistTransit is linked to negative SHAP values, indicating that increased distance from transit facilities suppresses running flow. UTAcc and NTL show positive associations at higher values. PopDen exhibits a dispersed pattern, with scattered positive contributions at high values but predominantly negative effects at moderate levels. LUM contributes positively with limited magnitude.

In the training configuration (Fig. 4e–f), SportDen overwhelmingly dominates, with high values consistently producing large positive SHAP contributions. In contrast, higher Continuity is associated with negative SHAP values. SurfFlat, Shade, and IntNum all show positive SHAP contributions at higher values, indicating supportive effects on training-oriented running flow.

#### Interpretation of configuration-dependent nonlinear associations

**Fig. 5:**
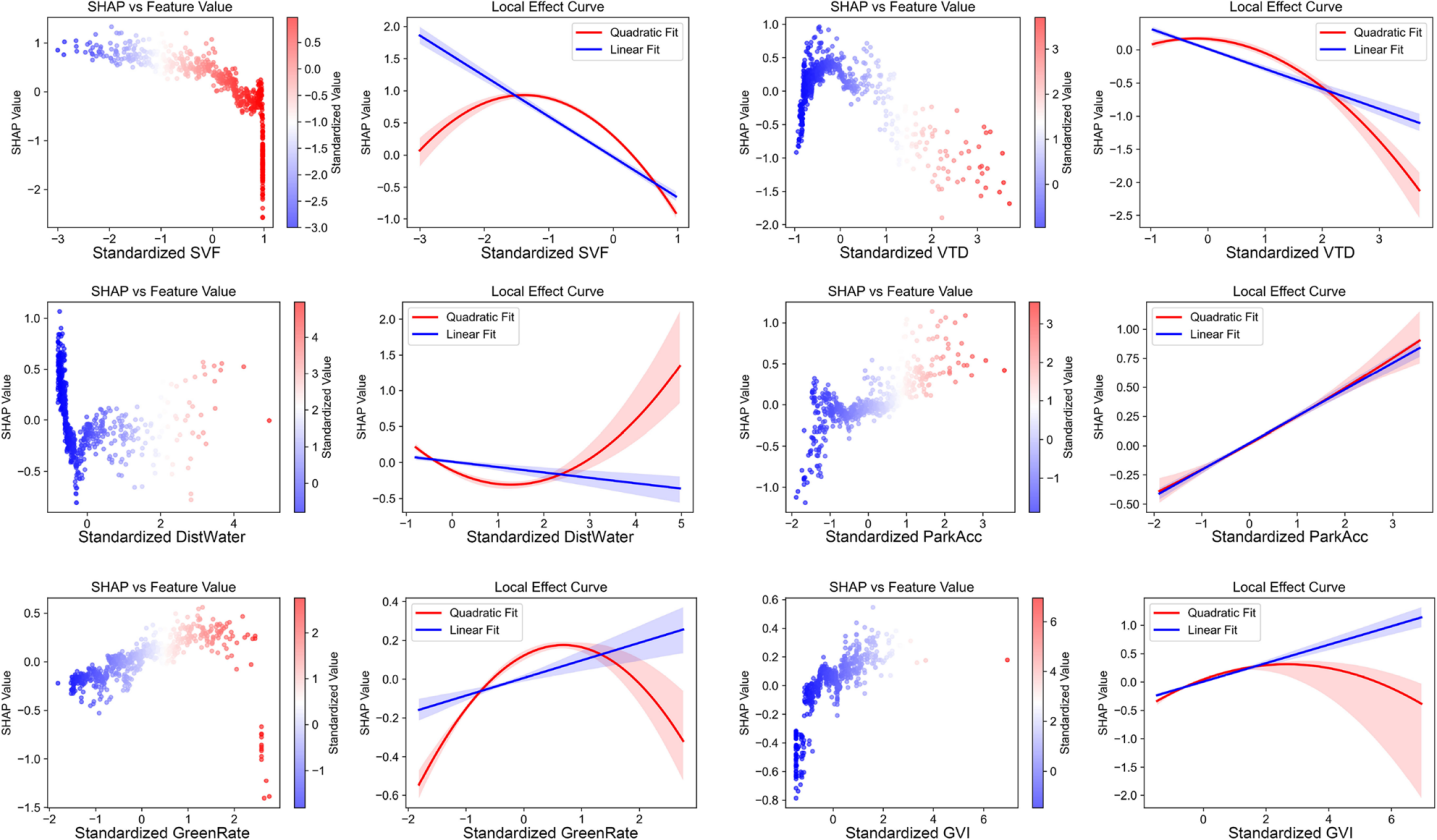
Non-linear associations between individual built-environment variables and running flow in Beijing during winter, spring, and summer 2024: first set of dependence plots based on Shapley additive explanations values. Each row displays the Shapley additive explanations (SHAP) dependence plot (left) and the corresponding local effect curve (right) for a given variable. The SHAP plot shows the marginal contribution of the standardised feature to the model output, with colour representing feature values. The right panel includes both linear and quadratic trend lines with 95% confidence intervals to illustrate non-linear patterns. Results are derived from global SHAP analysis using the full dataset

In the restorative running configuration, built environment variables exhibit distinct nonlinear response shapes in relation to running flow (Fig. 5). A group of variables, including SVF, VTD, GreenRate, and GVI, display clear inverted-U–shaped associations, with SHAP values increasing from low to moderate levels and declining once these variables exceed intermediate ranges, indicating the presence of optimal conditions rather than continuous enhancement. By contrast, DistWater shows a U-shaped relationship, characterised by positive contributions at shorter distances, a negative response at intermediate distances, and a gradual recovery at higher values. ParkAcc differs from these patterns and approximates a monotonic increasing association, with SHAP values rising steadily as accessibility improves. Together, these results indicate that restorative running flow is governed by multiple forms of nonlinearity, with different environmental attributes operating through distinct response structures.

**Fig. 6:**
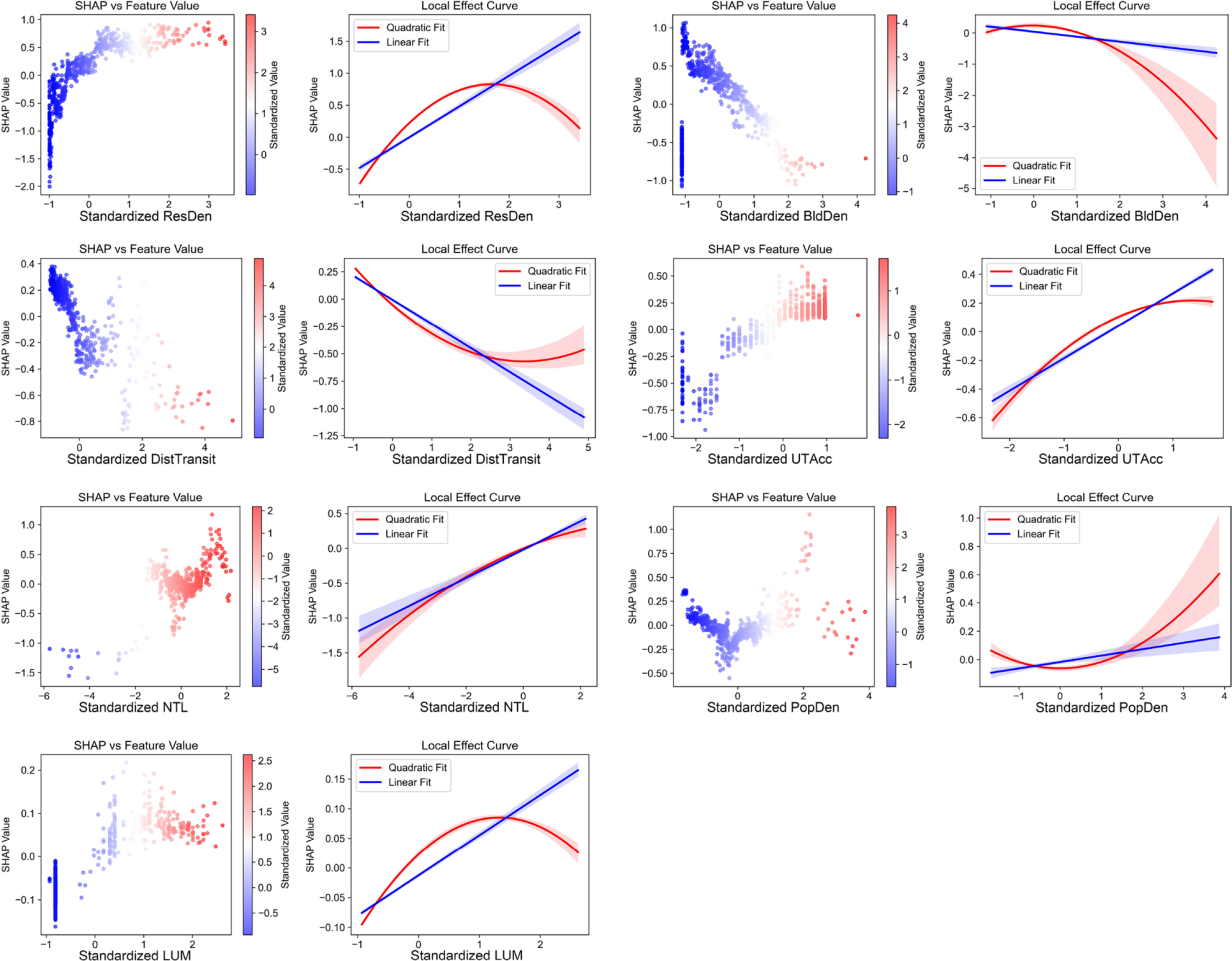
Non-linear associations between individual built-environment variables and running flow in Beijing during winter, spring, and summer 2024: second set of dependence plots based on Shapley additive explanations (SHAP) values

In the urban running configuration, built environment variables exhibit multiple nonlinear response structures in relation to running flow (Fig. 6). ResDen, BldDen, and LUM display clear inverted-U–shaped associations, with SHAP values increasing at lower to moderate levels and declining beyond intermediate ranges, indicating diminishing contributions at higher intensities. In contrast, DistTransit and PopDen show U-shaped relationships, characterised by negative contributions at intermediate values and more positive SHAP values at both lower and higher ends of their distributions. UTAcc and NTL exhibit asymmetric nonlinear patterns that approximate partial inverted-U structures. SHAP values increase over the observed range but show evident curvature and attenuation, deviating from a strictly monotonic response. Together, these results indicate that urban running flow is shaped by a mixture of complete and partial nonlinear structures, reflecting heterogeneous sensitivity to urban density, accessibility, and intensity-related attributes.

**Fig. 7:**
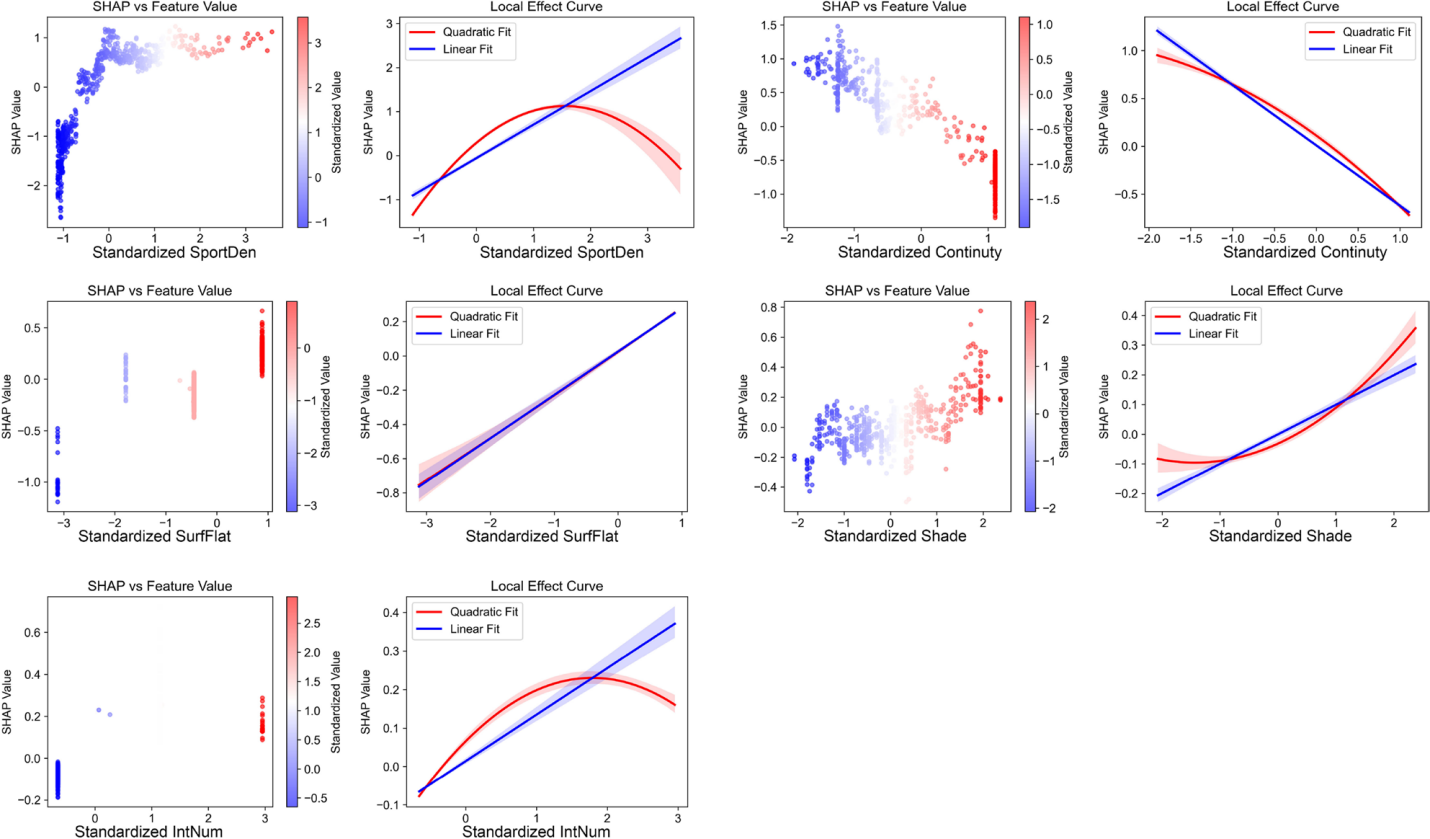
Non-linear associations between individual built-environment variables and running flow in Beijing during winter, spring, and summer 2024: third set of dependence plots based on Shapley additive explanations (SHAP) values

In the training running configuration, built environment variables display distinct nonlinear response structures in relation to running flow (Fig. 7). SportDen and IntNum exhibit clear inverted-U–shaped associations, with SHAP values increasing from low to moderate levels and declining once these variables exceed intermediate ranges, indicating diminishing contributions at higher intensities. Shade shows a U-shaped relationship, characterised by lower SHAP values at intermediate levels and more positive contributions toward both lower and higher ends of its distribution. Continuity presents an asymmetric nonlinear pattern that corresponds to the descending branch of an inverted-U structure, with SHAP values decreasing steadily across the observed range. In contrast, SurfFlat displays a predominantly monotonic increasing association, with SHAP values rising consistently as surface flatness improves. Together, these results highlight the coexistence of complete and partial nonlinear structures in shaping training-oriented running flow.

### Interaction effects of built environment variables across feature configurations

**Fig. 8:**
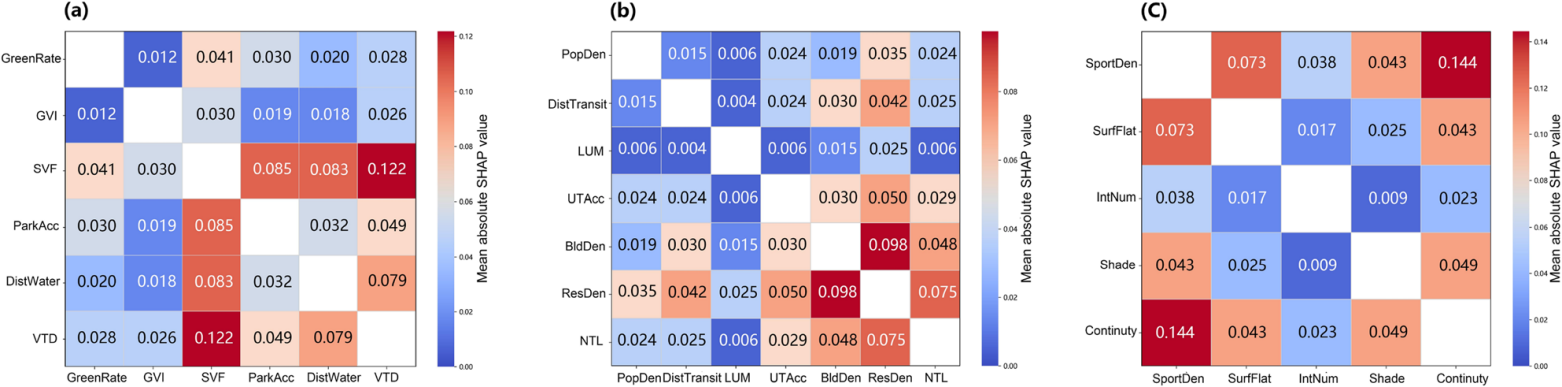
Interaction strength between built-environment variables across restorative, urban, and training running configurations in Beijing during winter, spring, and summer 2024, measured by mean absolute Shapley additive explanations (SHAP) interaction values. Panels show pairwise interaction strength, measured as mean absolute SHAP interaction values, for (**a**) the restorative, (**b**) the urban, and (**c**) the training running configurations. Values denote mean absolute SHAP interaction magnitudes on the model output scale and are not standardised. Interaction strength is therefore interpreted relatively, enabling comparison across feature pairs within the same configuration rather than across models

Figure 8 presents the interaction matrices of built environment variables across the restorative, urban, and training configurations. The matrices display mean absolute SHAP interaction values, reflecting the relative strength of pairwise interactions in shaping running flow within each configuration. In the restorative configuration (Fig. 8a), interaction effects are dominated by variables related to visual and experiential qualities. The strongest interactions involve SVF×VTD, SVF×ParkAcc, and SVF×DistWater, indicating that the contribution of visual openness is highly contingent on landscape diversity, accessibility, and proximity to water. Interactions among greenness indicators (GreenRate×GVI) are comparatively weak, suggesting limited synergistic effects beyond their individual contributions. In the urban configuration (Fig. 8b), interaction strength concentrates around density- and intensity-related variables. Notably strong interactions are observed between ResDen×BldDen, ResDen×UTAcc, and BldDen×NTL, highlighting that residential density, building intensity, and urban vitality jointly structure running flow in urban contexts. By contrast, interactions involving LUM remain relatively weak, indicating that land-use mix contributes primarily through main effects rather than interactive mechanisms. In the training configuration (Fig. 8c), interaction patterns are more focused and selective. The most prominent interaction is observed between SportDen×Continuity, followed by SportDen×SurfFlat and SportDen×Shade, indicating that the effect of specialised sports infrastructure is strongly modulated by route continuity and surface conditions. Interactions involving IntNum are comparatively modest, suggesting a secondary role in shaping training-oriented running flow.

**Fig. 9:**
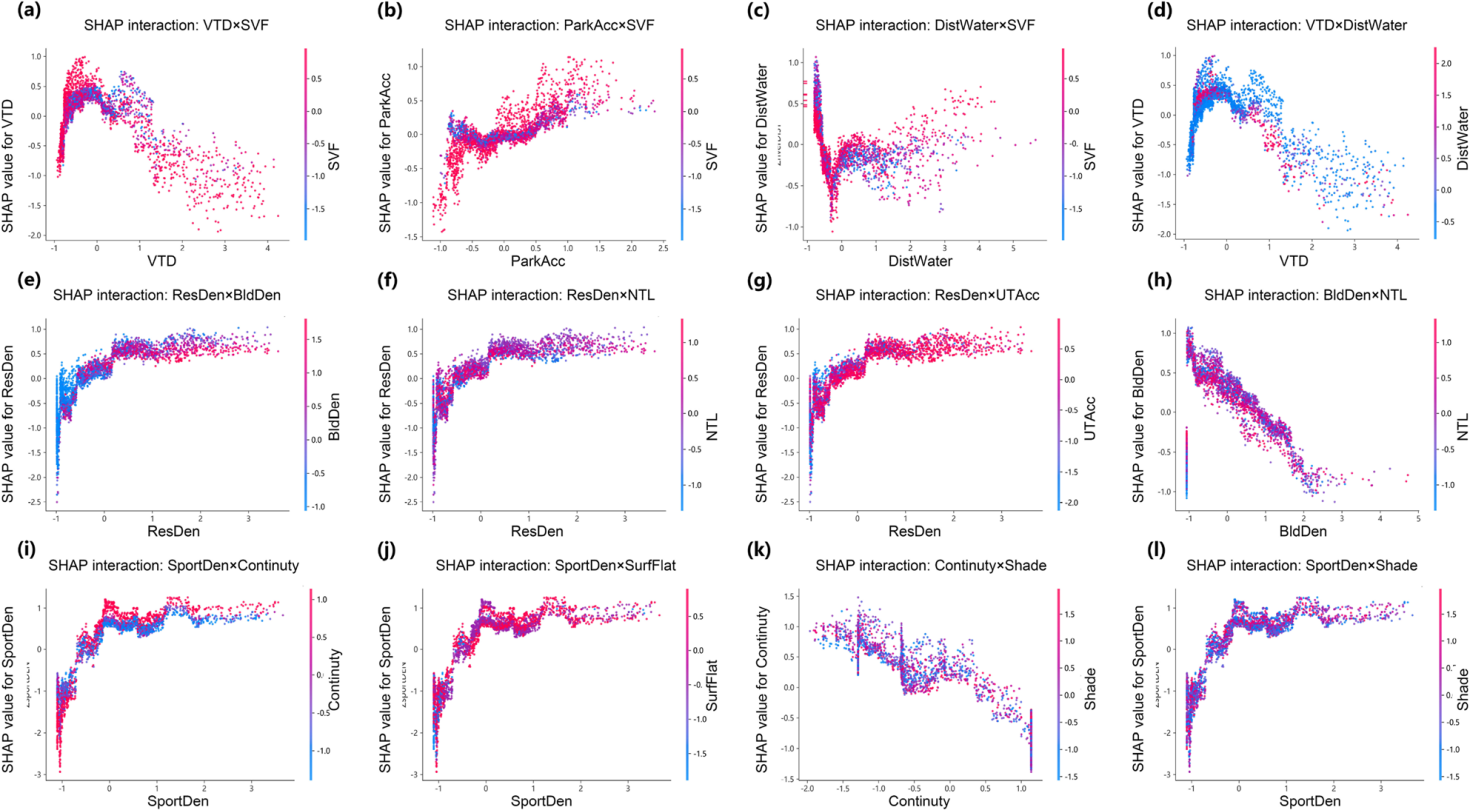
Pairwise interaction structures of built-environment variables across restorative, urban, and training running configurations in Beijing during winter, spring, and summer 2024. Dependence plots are based on Shapley additive explanations (SHAP) interaction values. Panels a–d show interaction structures in the restorative running configuration: (**a**) VTD × SVF, (**b**) ParkAcc × SVF, (**c**) DistWater × SVF, and (**d**) VTD × DistWater. Panels e–h show interaction structures in the urban running configuration: (**e**) ResDen × BldDen, (**f**) ResDen × NTL, (**g**) ResDen × UTAcc, and (**h**) BldDen × NTL. Panels i–l show interaction structures in the training running configuration: (**i**) SportDen × Continuity, (**j**) SportDen × SurfFlat, (**k**) Continuity × Shade, and (**l**) SportDen × Shade. In each panel, the x-axis denotes the focal feature and the y-axis denotes its SHAP contribution, with point colour indicating the interacting feature. Vertical colour stratification captures conditional, non-additive interaction structures rather than marginal effects. SHAP values are expressed on the model output scale and should be interpreted relatively within each configuration

In the restorative configuration, interaction effects are structured primarily by DistWater, VTD, and SVF (Fig. 9a–d). The DistWater×SVF interaction shows that SHAP contributions peak at low DistWater values, indicating that water-adjacent trails exert the strongest explanatory influence. Within this range, lower SVF is associated with higher SHAP values, while higher SVF corresponds to reduced contributions; this stratification reverses as DistWater increases, with higher SVF contributing more strongly at greater distances. The VTD×DistWater interaction further reveals a non-uniform pattern: near-water locations are associated with higher SHAP values at both low and high VTD levels, whereas mid-range VTD shows higher contributions farther from water. Together, these results indicate that vegetation structure effects are conditionally reweighted by water proximity rather than acting additively.

In the urban configuration, interaction effects are dominated by ResDen and its joint relationships with BldDen, UTAcc, and NTL (Fig. 9e–h). The ResDen×BldDen interaction shows that higher building density contributes more strongly at low residential density, with its influence weakening as ResDen increases. A similar pattern is observed for ResDen×UTAcc, where higher trail walk accessibility enhances SHAP values mainly in low-ResDen contexts, while its effect becomes muted at higher residential densities. In contrast, the BldDen×NTL interaction indicates that the negative contribution of building density intensifies under higher nighttime light levels, reflecting an antagonistic interaction. Overall, urban running flow is shaped by context-dependent modulation of density-related effects, rather than uniform structural influences.

In the training configuration, interaction effects centre on SportDen and its joint associations with Continuity, Shade, and SurfFlat (Fig. 9i–l). The SportDen×Continuity interaction exhibits clear stratification: at low SportDen, lower continuity is associated with higher SHAP values, whereas at moderate to high SportDen, higher continuity consistently contributes more strongly. The Continuity×Shade interaction is characterised by a dominant monotonic negative effect of continuity, with only weak stratification by shade. By contrast, the SportDen×Shade interaction shows pronounced differentiation at low SportDen, where higher shade corresponds to higher SHAP values, while this effect diminishes as SportDen increases. These patterns indicate configuration-specific interaction structures underlying training-oriented running flow.

## Discussion

### Rethinking built environment effects

Recent studies on the built environment’s influence on running behaviour have increasingly employed machine learning and interpretable methods to uncover non-linear marginal effects and threshold structures [12, 51]. Our findings align with prior research in several respects. For example, residential density exhibits a classic inverted U-shaped relationship—a pattern repeatedly confirmed by Liu et al. (2023) [17], Yang et al. (2025) [9], among others. This supports the behavioural mechanism of ‘optimal density’, wherein overly sparse areas lack the necessary agglomeration effects, while overly dense environments deter activity due to crowding and noise [33]. The consistency of this pattern across scales suggests a degree of structural robustness. However, our study also identifies deviations from established findings. Variables such as intersection density, building density, and land-use mix—typically associated with positive or inverted U-shaped effects—demonstrate monotonic or atypically decreasing trends in our analysis. This suggests that excessive spatial complexity or environmental interference may in fact dampen running activity. Notably, we observe a bimodal effect for distance to water. Both near-waterfront zones and distant forested areas exhibit higher running intensity than mid-range locations. This contrasts with Liu et al. (2023), who reported a negative association [17], and Yang et al. (2024), who found a positive one. Such results indicate that environmental variables do not function as isolated stimuli [14], but rather operate through configuration-dependent structural mechanisms.

Moving beyond individual variables, a growing body of research has explored interactions between built environment features to examine potential synergistic effects. For example, Yang et al. (2024a) adopted a scale-hierarchical strategy by pairing macro- and micro-level variables in extensive interaction modelling [14]. However, this approach is primarily grounded in spatial scale rather than perceptual coherence within behavioural contexts. As a result, structurally paired variables may not be simultaneously activated in individuals’ perception–decision–action chains, leading to counterintuitive or difficult-to-interpret results—such as the negative interaction between GVI and SDI. Subsequently, Yang et al. (2024) refined the method by selecting high-ranking SHAP features for interaction analysis [14]. While more streamlined in execution, this model-driven pathway construction lacked scenario-based validation of perceptual or functional synergy between variables, potentially limiting both explanatory depth and practical applicability. By contrast, we adopt a configuration-based analytical approach that explicitly models interaction structures among co-occurring built-environment features in relation to running behaviour. Variable pairings were selected based on theoretical assumptions of perceptual complementarity and behavioural co-activation. This allows for more contextually grounded interaction structures. For instance, SportDen×SurfFlat shows a strong positive interaction in the training model, reflecting dual demands for both facility density and ground quality among high-frequency runners. In the restorative model, ParkAcc×SVF exhibits an asymmetrical synergy, highlighting the combined restorative potential of park accessibility and sky openness under specific spatial configurations. Such behaviourally interpretable structures exemplify the distinctive advantages of context-driven interaction analysis.

In sum, this study shows that the behavioural influence of the built environment arises not from individual variables in isolation, but from structural affordances emerging from specific configurations of co-occurring features. Building on this insight, we introduce the concept of the Structural Affordance of the Built Environment to characterise how different variable configurations condition behavioural responses across contexts. This perspective moves beyond main-effect models and provides a configuration-based framework for interpreting interaction structures.

### Structural affordance of built environment

Affordance theory conceptualises behaviour as emerging from relational and situated interactions between individuals and their environments [23], rather than from isolated environmental attributes or internal intentions. Subsequent developments have emphasised the dynamic [22], socio-cultural [49], and situated nature of these relations [52], collectively implying that affordances are inherently configurational, arising from how multiple environmental cues co-occur and are perceived together within specific contexts. However, while this body of theory offers a rich conceptual account of relationality, it provides limited methodological guidance on how such multi-element relations can be empirically identified, compared, and evaluated at the population scale.

Empirical research on the built environment and physical activity has therefore tended to focus on marginal associations between individual variables and behaviour, or to rely on global models that aggregate heterogeneous relationships [53]. Although recent advances in interpretable machine learning, including SHAP-based approaches, allow non-linear and interactive effects to be detected [12, 13, 51], most applications remain variable-centric, emphasising relative importance rather than the configurational structures through which behavioural responses are organised. As demonstrated in our supplementary analysis (Supplementary Material S3), global models tend to foreground dominant predictors (e.g., SportDen, ResDen) while underrepresenting context-dependent interaction structures (e.g., VTD×SVF, SportDen×Continuity), indicating that methodological advances alone are insufficient without analytical designs that explicitly foreground configurational relations.

Against this backdrop, the concept of structural affordance of the built environment does not revise the philosophical premises of ecological or situated affordance theory, but extends its empirical scope and analytical resolution. Rather than focusing on momentary perception–action couplings, structural affordance shifts the unit of analysis to recurring and comparable configurational patterns through which affordances are stabilised and reproduced across populations and spatial settings. In this study, affordance is operationalised as a configuration-level empirical phenomenon, identified through the stability and transformation of interaction structures across alternative, theory-informed model specifications estimated on the same behavioural outcome. In doing so, the analysis reveals not isolated effects but coordinated environmental structures that consistently activate behaviour in complex urban contexts.

Across alternative feature configurations, our results reveal consistent and interpretable interaction patterns that function as empirical signatures of structural affordance. These patterns point to three mechanisms: pervasive marginal non-linearity, contextual modulation of effects across configurations, and synergistic structuring in which variables amplify or compensate for one another rather than acting additively. Together, these mechanisms demonstrate that the built environment operates not as a collection of independent features, but as a structured field of potential in which affordances are activated through coordinated configurations. This contribution lies in rendering relational and situated affordances empirically tractable at the population scale, enabling them to be identified, compared, and interpreted through interpretable non-linear modelling.

Finally, it should be noted that the behavioural data are derived from the Keep fitness app, whose users are predominantly younger and technologically engaged. Accordingly, the structural affordances identified here primarily reflect how built-environment configurations activate running behaviour within this population. Affordance manifestations may differ for other groups—such as older adults or less technologically connected populations—who may prioritise safety, surface quality, accessibility, or resting infrastructure. Structural affordance should therefore be interpreted as a population-contingent property of the built environment rather than a universal one, underscoring the need for future research across more diverse population groups.

### Policy implications

From a policy and urban governance perspective, the key contribution of this study is not the proposal of new spatial types or design paradigms, but a reframing of how environmental interventions are evaluated and prioritised in health-oriented planning. Although built-environment interventions are commonly implemented through multi-sectoral and integrated approaches [54], evidence building, investment prioritisation, and performance evaluation still rely largely on isolated indicators or checklist-based assessments of improvement [55]. Such approaches provide limited insight into whether multiple environmental elements jointly form behaviour-activating configurations that can be perceived and acted upon by users, helping to explain why similar interventions often produce divergent health outcomes across cities and neighbourhoods.

Empirical research has shown that the effects of built-environment attributes on physical activity vary substantially across urban contexts [56], and that improving single indicators does not reliably activate health behaviour. While recent governance studies have introduced multi-dimensional indicator systems and diagnostic matrices to support integrated decision-making, these tools typically remain normative and offer limited empirical insight into how environmental elements interact to generate behavioural responses.

In this context, structural affordance is defined as a goal-oriented diagnostic framework for environmental configurations, rather than a prescriptive design model. It shifts planning decisions from uniform indicator optimisation toward identifying the minimum viable environmental configuration required to activate a specific health behaviour. The central policy question thus becomes whether critical interactions among environmental elements are jointly present, or whether missing interactions undermine the formation of a complete behaviour-activating configuration. This configurational perspective supports planning and public investment decisions in three ways. First, it enables the diagnosis of areas where multiple elements exist but fail to operate synergistically, helping to explain unstable intervention effects. Second, under budget constraints, it guides prioritisation toward repairing key interaction gaps rather than uniformly upgrading all indicators. Third, in plan comparison and implementation assessment, it offers a shared criterion—whether a stable behaviour-activating configuration has been formed—reducing the risk that sector-specific targets are met without producing integrated health outcomes.

By shifting health-oriented planning from identifying influential single factors to assessing the robustness of environmental configurations across explanatory conditions, this study provides a mechanism-based explanation for policy outcome heterogeneity. This configuration-oriented perspective complements, rather than replaces, existing comprehensive planning practices by offering a more interpretable and operational diagnostic language for evidence-based health governance.

### Limitation

While this study offers theoretical and methodological advances, several limitations should be acknowledged. First, running trajectories were obtained from the Keep platform, which predominantly represents younger, working-age users. The findings should therefore be interpreted as environment–behaviour associations at the trail-segment level rather than population-level prevalence estimates. Nevertheless, younger cohorts constitute the most active running population in Beijing, and prior studies show that crowdsourced fitness data reliably capture fine-grained spatial patterns of recreational activity despite demographic skews. Second, although multiple seasonal months were included, the analysis relies on a limited set of temporal snapshots and does not explicitly represent extreme temperature conditions or severe air pollution episodes. Longer continuous time series would be required to assess the temporal stability of configuration-dependent interaction structures under more extreme environmental contexts. Third, physical environmental exposures such as air pollution and noise were not explicitly modelled [57]. The omission of these factors may partially confound estimated built-environment effects, and future work integrating physical exposure data could further examine how environmental structures and physical stressors jointly condition running behaviour. Finally, the use of grid-based spatial units may introduce spatial autocorrelation between adjacent cells due to trail continuity and unobserved spatial processes. Although residual dependence is substantially reduced after controlling for built-environment features, some spatial structure may remain. As this study focuses on identifying interaction structures rather than estimating spatial spillover effects, future research could incorporate explicit spatial modelling frameworks to further disentangle configuration effects from spatial dependence.

## Conclusions

This study examined how urban environments shape running behaviour through both temporal rhythms and spatial structures. The findings reveal clear morning–evening peaks, an inverted-U weekly pattern, and a concentric spatial distribution concentrated in central areas and continuous spaces such as waterfronts and forest sports parks. Beyond these regularities, the analysis shows that built-environment influences are structured through configuration-specific interactions rather than independent or additive effects. Three dominant interaction configurations are identified: visual–landscape structures combining vegetation texture, sky openness, and water proximity; urban density configurations linking residential intensity, building form, accessibility, and nightlight intensity; and training configurations integrating sports facility density, urban trails continuity, surface flatness, and shading. These results indicate that running behaviour is shaped by coordinated built-environment structures rather than isolated variables.

## Supplementary Information

Supplementary Material 1.

## Data Availability

The datasets analysed during the current study are available from the corresponding author on reasonable request.

## Abbreviations

GPS: Global Positioning System
SHAP: Shapley Additive Explanations
PDP: Partial dependence plot
TAZs: Traffic Analysis Zones
POI: Point of Interest
AOI: Area of Interest
LightGBM: Light Gradient Boosting Machine
