# Supplementary material for: How urban environments structure running behaviour in Beijing during winter, spring, and summer 2024: spatiotemporal patterns and configuration-specific interactions from running trajectory data
Source: Arch Public Health. 2026 Apr 27;84:131. doi: 10.1186/s13690-026-01919-x (PMC13267637; doi:10.1186/s13690-026-01919-x)
Supplement: Supplementary file 1 — Supplementary Material 1. [file 13690_2026_1919_MOESM1_ESM.docx]

**Supplementary Material**

# **S1 Multicollinearity Diagnostics of Built-Environment Variables**

This table reports variance inflation factors (VIFs) for all built-environment variables. All VIF values are below 2.5, indicating no problematic multicollinearity and supporting reliable interpretation of individual and interaction effects.

**Table S1** Multicollinearity diagnostics for built-environment variables used to analyse running behaviour in Beijing during winter, spring, and summer 2024.

| Variable | Variance inflation factor | Variable | Variance inflation factor |
| --- | --- | --- | --- |
| **Population density (PopDen)** | 1.559 | **Sky View Factor (SVF)** | 2.497 |
| **Residential Density (ResDen)** | 2.403 | **Surface Flatness (SurfFlat)** | 1.294 |
| **Building Density (BldDen)** | 1.917 | **Urabn Trails Accessibility (UTAcc)** | 1.349 |
| **Indoor Sports Facility Density (SportDen)** | 1.792 | **Park Accessibility (ParkAcc)** | 1.250 |
| **Land Use Mix (LUM)** | 1.946 | **Number of Intersections (IntNum)** | 1.093 |
| **Vegetation Type Diversity (VTD)** | 1.354 | **Distance to Metro/Bus/Parking Entrance (DistTransit)** | 1.818 |
| **Green Coverage Ratio (GreenRate)** | 1.422 | **Distance to Waterway (DistWater)** | 1.105 |
| **Green View Index (GVI)** | 1.089 | **Night-time light intensity (NTL)** | 1.417 |
| **Shading Ratio (Shade)** | 1.030 | **Urban Trail Continuity (Continuity)** | 1.371 |

# **S2 Scale Sensitivity Analysis of Grid-Based Spatial Units**

To assess the robustness of scale choice, we replicated the full modelling and SHAP analysis using a coarser 500-m grid (Fig. S1). Across all three running configurations, the relative importance ranking of built-environment variables remains highly consistent with the main analysis based on the baseline grid resolution.

In the restorative configuration (Fig. S1a–b), SVF, VTD, and distance to water continue to dominate feature contributions, and their SHAP value distributions preserve the same directional patterns and dispersion structure observed in the main results. Minor compression of SHAP ranges is observed, reflecting spatial aggregation at a coarser scale, but without altering the relative influence of key green–blue environment variables.

For the urban configuration (Fig. S1c–d), residential density and building density remain the two most influential predictors, followed by transit accessibility and urban trail accessibility. The sign and spread of SHAP values are stable, indicating that density- and accessibility-driven effects are not sensitive to moderate changes in spatial resolution.

Similarly, in the training configuration (Fig. S1e–f), sport facility density consistently exhibits the largest contribution, with continuity and surface flatness retaining secondary importance. While the absolute magnitude of SHAP values is slightly reduced at the 500-m scale, the dominance of sport-oriented and performance-related features is preserved.

Overall, the sensitivity analysis confirms that the identified importance structure and feature effect patterns are robust to grid-scale variation. Scale aggregation primarily affects the magnitude of SHAP values rather than the ordering or qualitative interpretation of environmental influences, supporting the stability of the main findings.

| 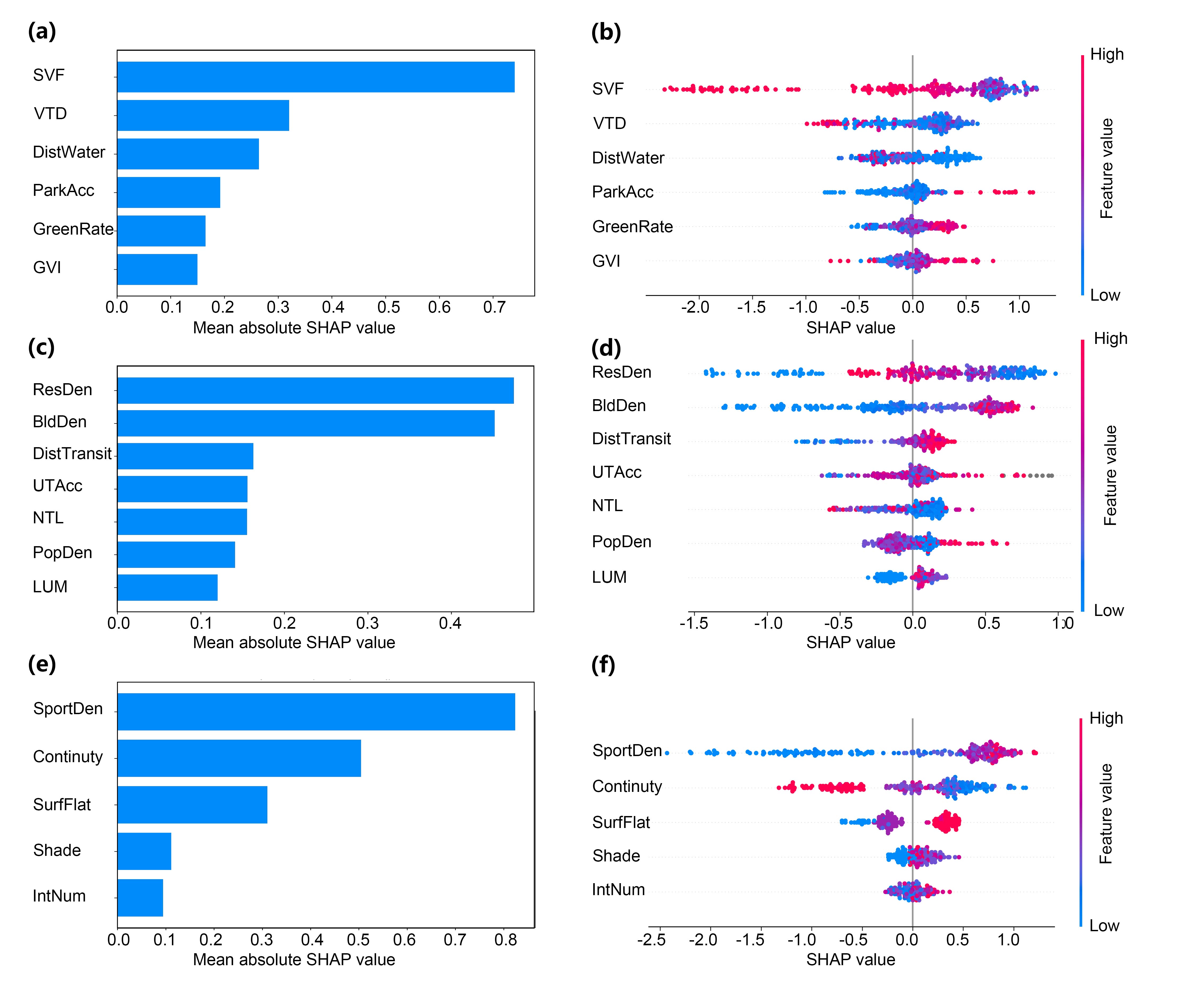 |
| --- |
| **Fig. S1** Relative importance of built-environment variables and distribution of Shapley additive explanations (SHAP) values at the 500-m grid scale in Beijing during winter, spring, and summer 2024. **(a)** Mean absolute SHAP values reflecting the overall contribution of each variable to the model prediction. **(b)** SHAP summary plot illustrating the direction and magnitude of each variable’s effect. Warmer colours indicate higher feature values; cooler colours indicate lower values. |

Fig. S2 presents SHAP interaction dependence plots derived from the 500-m grid, corresponding to the strongest interaction pairs identified in the baseline analysis. Overall, the interaction structures observed at the finer resolution are largely preserved, indicating that the identified configuration-dependent relationships are robust to spatial aggregation.

In the restorative configuration (Fig. S2a–d), interactions involving SVF, VTD, and distance to water retain their characteristic non-linear and conditional patterns. In particular, the interaction between distance to water and SVF continues to show stratified SHAP contributions at short distances, with stronger explanatory power concentrated near waterfront trails, while SVF-related colour separation becomes more diffuse as distance increases. Similarly, the VTD × distance-to-water interaction preserves its asymmetric pattern, with proximity to water remaining influential at both low and high VTD levels.

For the urban configuration (Fig. S2e–h), the interaction structure centred on residential density remains highly stable. Interactions between residential density and building density, nighttime light intensity, and urban trail accessibility continue to exhibit consistent monotonic and saturating patterns. The interaction between building density and nighttime light maintains its negative gradient, suggesting persistent coupling between built intensity and urban activity context across scales.

In the training configuration (Fig. S2i–l), sport facility density continues to dominate interaction effects. Its interactions with continuity, surface flatness, and shading display similar conditional patterns to those observed in the main analysis, with minor attenuation in SHAP magnitude. The interaction between continuity and shading retains a clear negative trend in continuity effects, while colour stratification by shading remains comparatively weak, indicating limited modification by shading at the aggregated scale.

Across all configurations, increasing the grid size primarily reduces the dispersion and absolute magnitude of SHAP interaction values but does not alter the relative ordering, directional patterns, or qualitative interpretation of interaction mechanisms. These results confirm that the identified interaction structures reflect stable spatial relationships rather than artefacts of grid resolution.

| 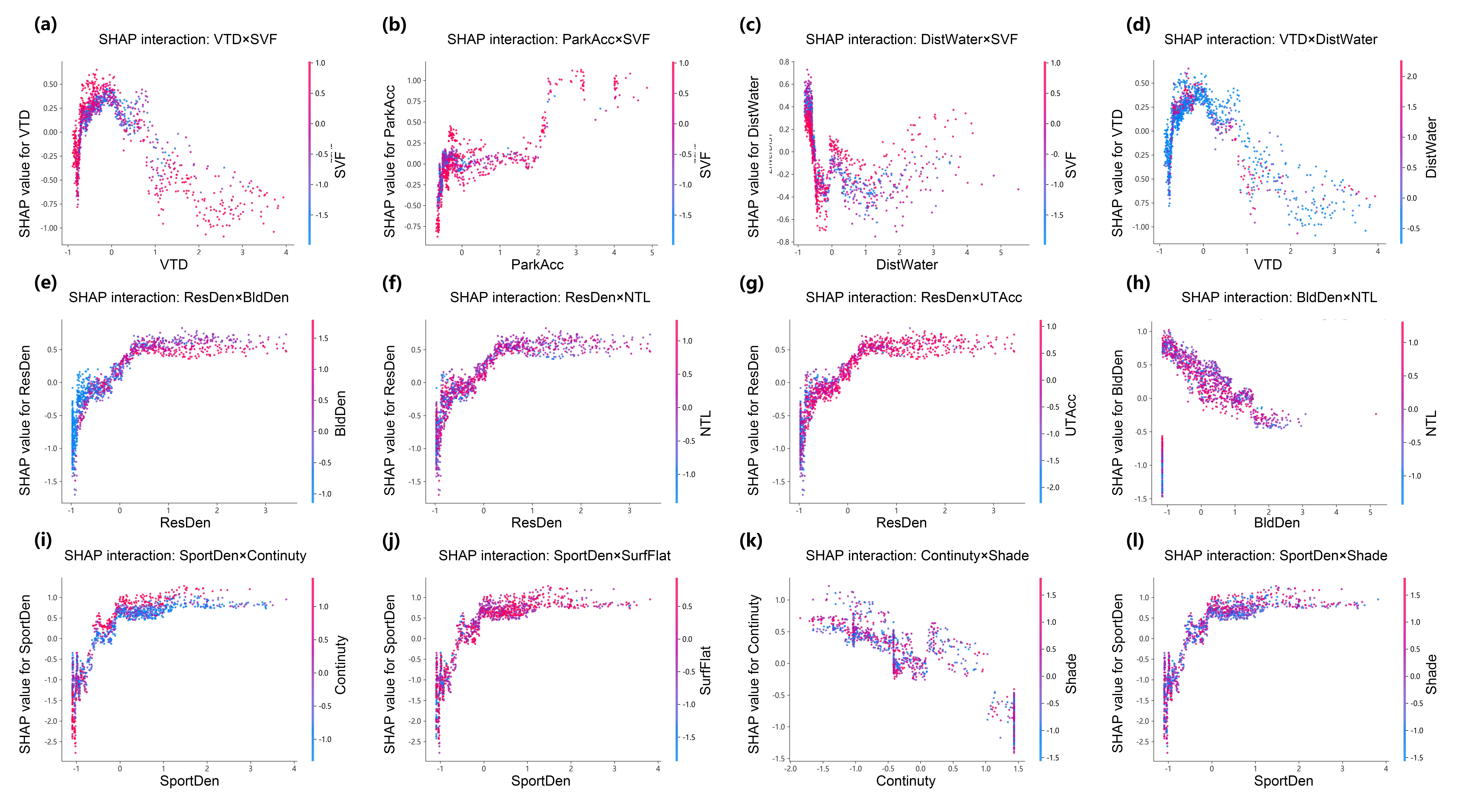 |
| --- |
| **Fig. S2** Pairwise interaction structures of built-environment variables at the 500-m grid scale in Beijing during winter, spring, and summer 2024: dependence plots based on Shapley additive explanations (SHAP) interaction values. The x-axis denotes the focal feature and the y-axis its SHAP contribution, with colour indicating the interacting feature. Vertical colour stratification captures conditional, non-additive interaction structures rather than marginal effects. SHAP values are expressed on the model output scale and interpreted relatively within each configuration. |

# **S3 Comparison between Configurational Specifications and the Fully Specified Model**

Fig. S3(a) presents the global SHAP importance ranking, and Fig. S3(b) shows the corresponding SHAP value distributions. The fully specified model is characterised by a pronounced concentration of explanatory power in a small subset of predictors, most notably sport facility density (SportDen) and urban trail continuity. Their SHAP magnitudes substantially exceed those of all other variables, indicating strong dominance of marginal effects in the aggregated specification.

| 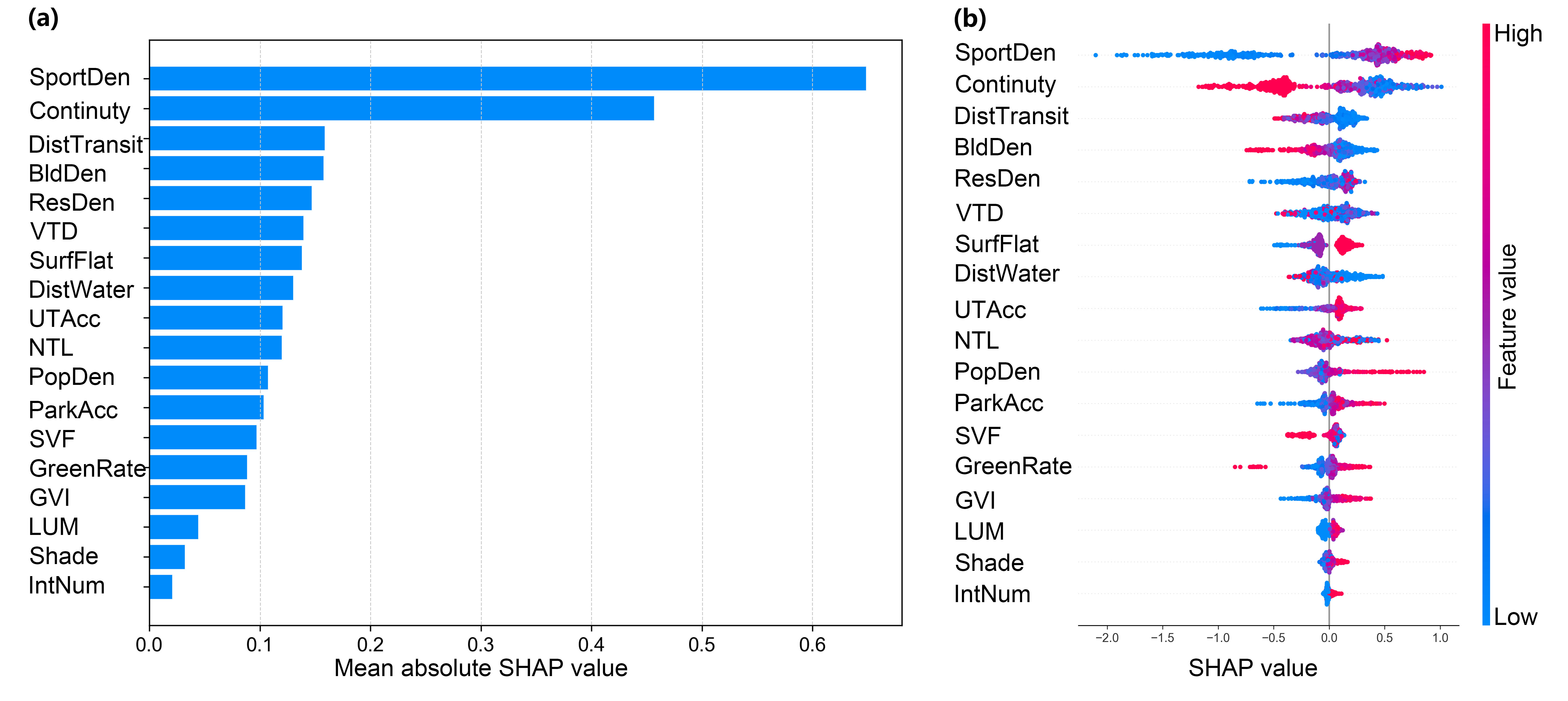 |
| --- |
| **Fig. S3** Relative importance of built-environment variables and distribution of Shapley additive explanations (SHAP) values from the fully specified model in Beijing during winter, spring, and summer 2024. **(a)** Mean absolute SHAP values reflecting the overall contribution of each variable to the model prediction. **(b)** SHAP summary plot illustrating the direction and magnitude of each variable’s effect in the fully specified model. Warmer colours indicate higher feature values; cooler colours indicate lower values. |

We further examined SHAP interaction patterns in the fully specified model. The top eight interaction pairs ranked by mean absolute SHAP interaction values are visualised in Fig. S4. These interactions are overwhelmingly concentrated in pairs involving SportDen, closely reflecting the global importance hierarchy. As a result, interaction salience in the full model largely mirrors marginal feature dominance, offering limited insight into context-dependent or mechanism-specific interaction structures.

| 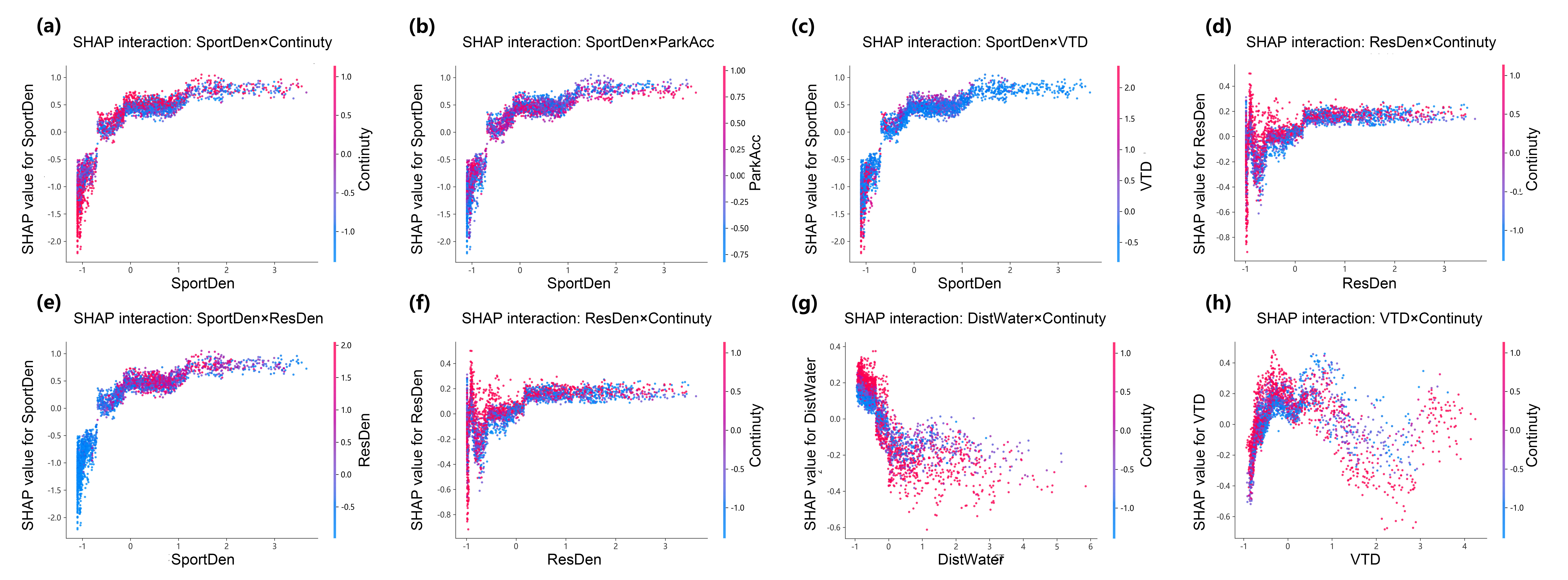 |
| --- |
| **Fig. S4** Dominant pairwise interaction structures identified in the fully specified model in Beijing during winter, spring, and summer 2024. |

These results contrast sharply with the interaction patterns obtained from the three configurational specifications (Restorative, Urban, and Training). For each specification, theoretically relevant interaction pairs were examined using SHAP interaction dependence plots (Fig. S5). Compared with the fully specified model, the configurational specifications exhibit more coherent point distributions and clearer monotonic or non-linear trends, indicating interaction structures that are more interpretable and internally consistent within each behavioural context.

| 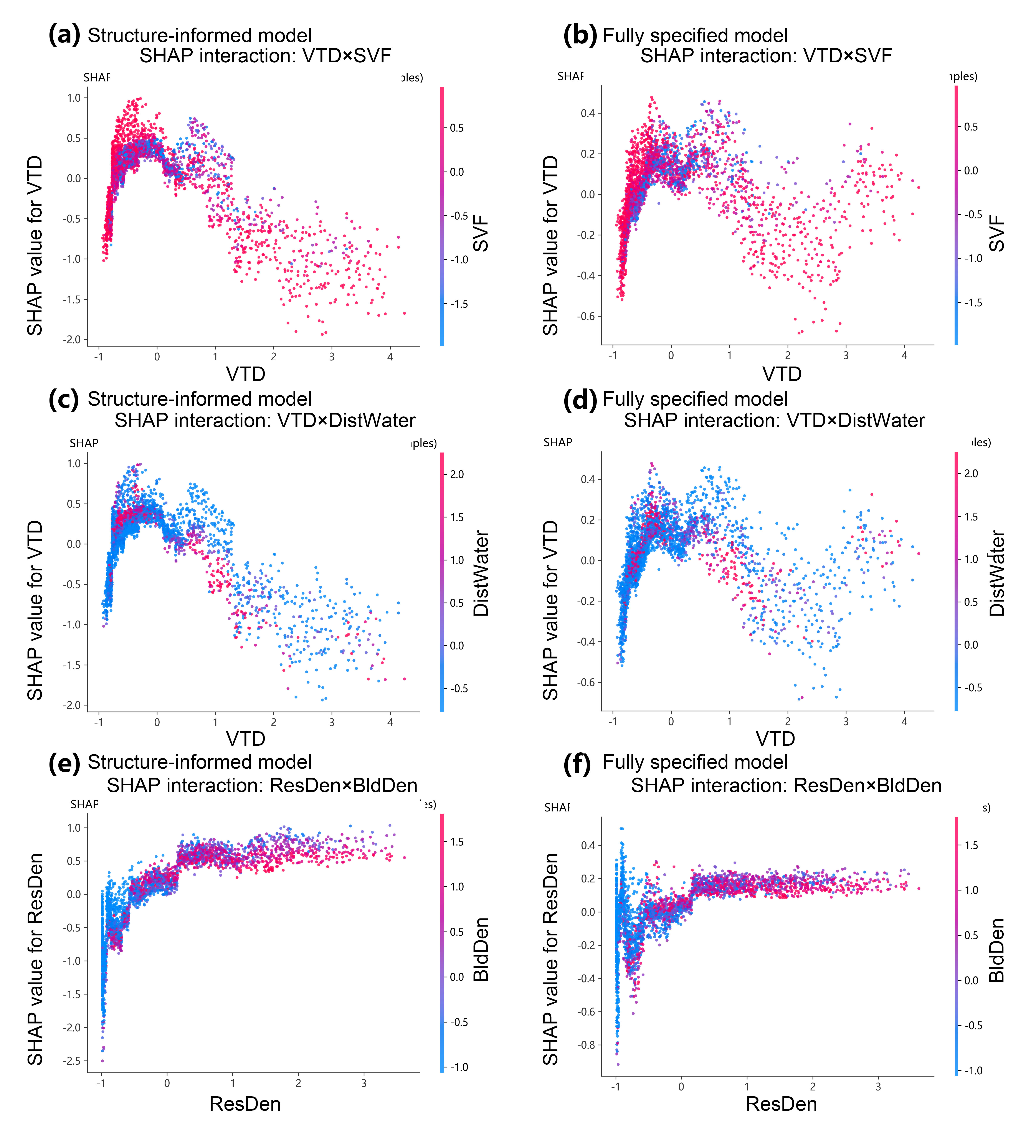 |
| --- |
| **Fig. S5** Comparison of pairwise interaction structures between the fully specified model and the configurational specifications used to analyse running behaviour in Beijing during winter, spring, and summer 2024. |

To quantitatively support this comparison, Supplementary Table S2 reports predictive performance metrics (R², RMSE, MAE), importance concentration indices (Top-k share, Gini coefficient, and Herfindahl–Hirschman Index), and interaction stability measures (Jaccard similarity and Spearman rank correlation). While the fully specified model achieves the highest predictive accuracy (R² = 0.568), it also exhibits the strongest concentration of explanatory power. By contrast, all configurational specifications substantially reduce dominance concentration (lower Gini and HHI values) and produce stable and reproducible interaction rankings. Importantly, several theoretically meaningful interactions—such as SVF×VTD and SVF×ParkAcc—do not appear among the top-ranked interactions in the fully specified model across repeated runs. These interactions, however, consistently emerge in the Restorative configurational specification, while remaining selectively absent in the Urban and Training specifications. Interaction rankings in the configurational models are at least as stable as those of the fully specified model (Jaccard similarity: 0.94–1.00; Spearman correlation≈0.98).

**Table S2** Comparative performance, importance concentration, and interaction stability metrics across model configuration

| Model configuration | Variables | R² | RMSE | MAE | Top1 share | Top3 share | Top5 share | HHI | Gini | Jaccard (Top-20) | Spearman |
| --- | --- | --- | --- | --- | --- | --- | --- | --- | --- | --- | --- |
| Fully specified | 18 | 0.568 | 1.394 | 0.920 | 0.104 | 0.289 | 0.449 | 0.069 | 0.283 | 0.900 | 0.980 |
| The Restorative configuration | 6 | 0.438 | 1.616 | 1.108 | 0.214 | 0.589 | 0.878 | 0.174 | 0.113 | 1.000 | 0.981 |
| The Urban configuration | 7 | 0.437 | 1.540 | 1.101 | 0.268 | 0.606 | 0.871 | 0.176 | 0.262 | 0.939 | 0.987 |
| The Training configuration | 5 | 0.430 | 1.683 | 1.109 | 0.377 | 0.851 | 1.000 | 0.267 | 0.320 | 1.000 | 0.991 |

*Note: R², coefficient of determination; RMSE, root mean square error; MAE, mean absolute error; Top1 share, importance share of the most influential variable; Top3 share, cumulative importance share of the three most influential variables; Top5 share, cumulative importance share of the five most influential variables; HHI, Herfindahl–Hirschman Index; Gini, Gini coefficient; Jaccard (Top-20), Jaccard similarity coefficient based on the top 20 variables; Spearman, Spearman rank correlation coefficient.*
